# *In Vivo* Thermodynamic Analysis of Glycolysis in Clostridium thermocellum and Thermoanaerobacterium saccharolyticum Using ^13^C and ^2^H Tracers

**DOI:** 10.1128/mSystems.00736-19

**Published:** 2020-03-17

**Authors:** Tyler B. Jacobson, Travis K. Korosh, David M. Stevenson, Charles Foster, Costas Maranas, Daniel G. Olson, Lee R. Lynd, Daniel Amador-Noguez

**Affiliations:** a Center for Bioenergy Innovation, Oak Ridge National Laboratory, Oak Ridge, Tennessee, USA; b Department of Bacteriology, University of Wisconsin–Madison, Madison, Wisconsin, USA; c Department of Chemical Engineering, The Pennsylvania State University, University Park, Pennsylvania, USA; d Thayer School of Engineering, Dartmouth College, Hanover, New Hampshire, USA; State University of Maringá

**Keywords:** microbial metabolism, metabolic flux analysis, MFA, mass spectrometry, biofuels, Gibbs free energy, stable isotope tracers, thermophilic bacteria, *Clostridium thermocellum*, metabolic flux, isotope tracers

## Abstract

Clostridium thermocellum and Thermoanaerobacterium saccharolyticum are thermophilic anaerobic bacteria with complementary metabolic capabilities that utilize distinct glycolytic pathways for the conversion of cellulosic sugars to biofuels. We integrated quantitative metabolomics with ^2^H and ^13^C metabolic flux analysis to investigate the *in vivo* reversibility and thermodynamics of the central metabolic networks of these two microbes. We found that the glycolytic pathway in *C. thermocellum* operates remarkably close to thermodynamic equilibrium, with an overall drop in Gibbs free energy 5-fold lower than that of *T. saccharolyticum* or anaerobically grown Escherichia coli. The limited thermodynamic driving force of glycolysis in *C. thermocellum* could be attributed in large part to the small free energy of the phosphofructokinase reaction producing fructose bisphosphate. The ethanol fermentation pathway was also substantially more reversible in *C. thermocellum* than in *T. saccharolyticum*. These observations help explain the comparatively low ethanol titers of *C. thermocellum* and suggest engineering interventions that can be used to increase its ethanol productivity and glycolytic rate. In addition to thermodynamic analysis, we used our isotope tracer data to reconstruct the *T. saccharolyticum* central metabolic network, revealing exclusive use of the Embden-Meyerhof-Parnas (EMP) pathway for glycolysis, a bifurcated tricarboxylic acid (TCA) cycle, and a sedoheptulose bisphosphate bypass active within the pentose phosphate pathway.

**IMPORTANCE** Thermodynamics constitutes a key determinant of flux and enzyme efficiency in metabolic networks. Here, we provide new insights into the divergent thermodynamics of the glycolytic pathways of *C. thermocellum* and *T. saccharolyticum*, two industrially relevant thermophilic bacteria whose metabolism still is not well understood. We report that while the glycolytic pathway in *T. saccharolyticum* is as thermodynamically favorable as that found in model organisms, such as E. coli or Saccharomyces cerevisiae, the glycolytic pathway of *C. thermocellum* operates near equilibrium. The use of a near-equilibrium glycolytic pathway, with potentially increased ATP yield, by this cellulolytic microbe may represent an evolutionary adaptation to growth on cellulose, but it has the drawback of being highly susceptible to product feedback inhibition. The results of this study will facilitate future engineering of high-performance strains capable of transforming cellulosic biomass to biofuels at high yields and titers.

## Abstract (precis)

Thermodynamics constitutes a key determinant of flux and enzyme efficiency in metabolic networks. Here, we provide new insights into the divergent thermodynamics of the glycolytic pathways of *C. thermocellum* and *T. saccharolyticum*, two industrially relevant thermophilic bacteria whose metabolism still is not well understood. We report that while the glycolytic pathway in *T. saccharolyticum* is as thermodynamically favorable as that found in model organisms, such as E. coli or Saccharomyces cerevisiae, the glycolytic pathway of *C. thermocellum* operates near equilibrium. The use of a near-equilibrium glycolytic pathway, with potentially increased ATP yield, by this cellulolytic microbe may represent an evolutionary adaptation to growth on cellulose, but it has the drawback of being highly susceptible to product feedback inhibition. The results of this study will facilitate future engineering of high-performance strains capable of transforming cellulosic biomass to biofuels at high yields and titers.

## INTRODUCTION

Thermoanaerobacterium saccharolyticum and Clostridium thermocellum are thermophilic, anaerobic bacteria with complementary metabolic capabilities being developed for industrial-scale production of biofuels, such as ethanol, from lignocellulosic biomass (1–3). *C. thermocellum* readily solubilizes lignocellulosic biomass and ferments cellulose-derived sugars, including oligomers, to ethanol, acetate, lactate, formate, and H_2_. However, *C. thermocellum* is incapable of utilizing the hemicellulose fraction of biomass (containing xylose, arabinose, mannose, and galactose) (4, 5). Metabolic engineering efforts have increased the native ethanol yields and titers of *C. thermocellum*, but the best strains, with a titer of ∼25 g/liter and 75% theoretical yield, do not yet match the productivity of noncellulolytic ethanologens such as Saccharomyces cerevisiae and Zymomonas mobilis (6–9). In contrast, *T. saccharolyticum* cannot solubilize lignocellulosic biomass or cellulose but readily ferments hemicellulose-derived sugars, including oligomers, to ethanol, acetate, lactate, and H_2_ (4, 10). *T. saccharolyticum* has been engineered to produce ethanol at greater than 90% theoretical yields and at titers of up to 70 g/liter (3, 10). These two thermophilic bacteria have been used in cocultures that seek to combine the cellulolytic capability of *C. thermocellum* with the higher ethanol productivity and hemicellulose-consuming capability of *T. saccharolyticum* (11). Several previous studies have directly compared the fermentation capabilities of *C. thermocellum* and *T. saccharolyticum*, and *T. saccharolyticum* has been used frequently as a source of genes to engineer enhanced ethanol productivity in *C. thermocellum* (12–19).

**FIG 1:**
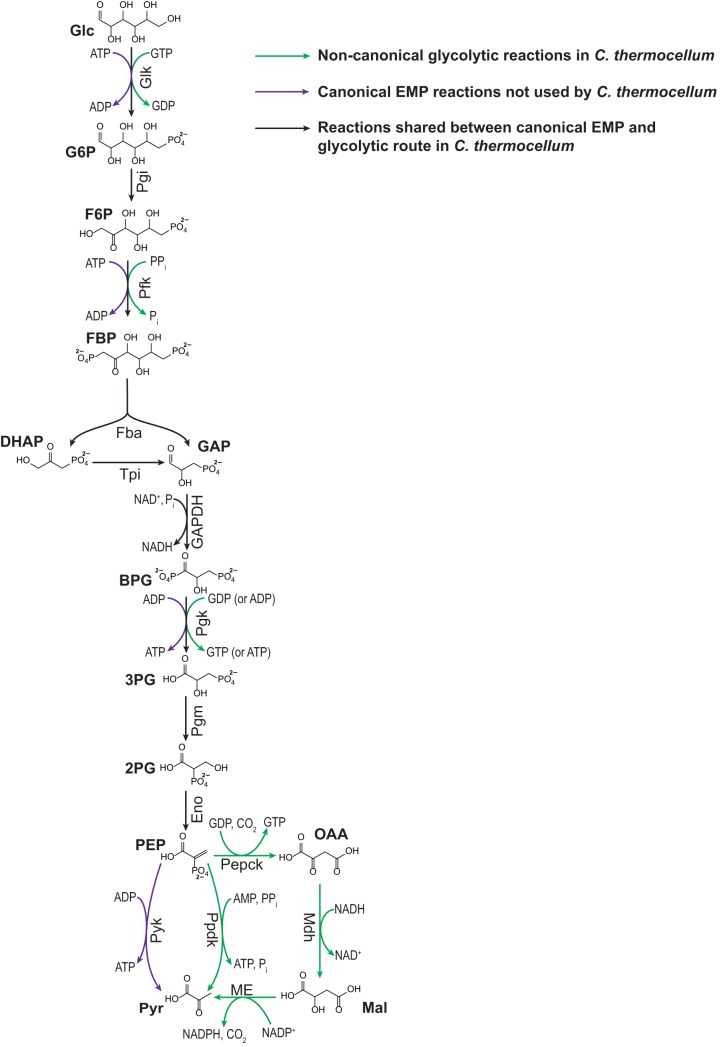
Glycolytic pathway of *C. thermocellum. C. thermocellum* uses a glycolytic pathway that differs from the canonical EMP pathway. Black arrows represent reactions that are common between EMP glycolysis and *C. thermocellum* glycolysis. Green arrows represent reactions unique to glycolysis in *C. thermocellum*. Purple arrows represent EMP glycolytic reactions not present in *C. thermocellum*. Abbreviations: 2PG, 2-phosphoglycerate; 3PG, 3-phosphoglycerate; BPG, 1,3-bisphosphoglycerate; DHAP, dihydroxyacetone phosphate; EMP, Embden-Meyerhof-Parnas pathway; Eno, phosphopyruvate hydratase; F6P, fructose-6-phosphate; Fba, fructose-1,6-bisphosphate aldolase; FBP, fructose-1,6-bisphosphate; G6P, glucose-6-phosphate; GAP, glyceraldehyde-3-phosphate; GAPDH, glyceraldehyde-3-phosphate dehydrogenase; Glc, glucose; Glk, glucokinase; Mal, malate; Mdh, malate dehydrogenase; ME, malic enzyme; OAA, oxaloacetate; PEP, phosphoenolpyruvate; Pepck, phosphoenolpyruvate carboxykinase; Pfk, phosphofructokinase; Pgi, phosphoglucose isomerase; Pgk, phosphoglycerate kinase; Pgl, phosphogluconolactonase; Pgm, phosphoglycerate mutase; Ppdk, pyruvate phosphate dikinase; PPi, pyrophosphate; Pyk, pyruvate kinase; Pyr, pyruvate; Tpi, triose-phosphate isomerase.

In addition to the differences outlined above, *T. saccharolyticum* and *C. thermocellum* possess distinct glycolytic pathways. While genome annotation suggests that *T. saccharolyticum* uses the canonical Embden-Meyerhof-Parnas (EMP) pathway, the glycolytic pathway in *C. thermocellum* has been shown to possess several unique characteristics (18, 20–23) (Fig. 1). Most notably, the conversion of fructose-6-phosphate (F6P) to fructose-1,6-bisphosphate (FBP), catalyzed by phosphofructokinase (Pfk), is coupled to pyrophosphate (PP_i_) rather than ATP hydrolysis (20, 24, 25). In addition, glucokinase (Glk; Glc + NTP→G6P) utilizes GTP instead of ATP as the high-energy phosphate donor, and phosphoglycerate kinase (Pgk; BPG + NDP → 3PG + NTP) is capable of producing both ATP and GTP (20, 26). Finally, *C. thermocellum* lacks a pyruvate (Pyr) kinase (Pyk; PEP + ADP→Pyr + ATP) and instead converts phosphoenolpyruvate (PEP) to Pyr via one of two routes: (i) pyruvate phosphate dikinase (Ppdk; PEP + AMP + PP_i_→Pyr + ATP + P_i_) and (ii) the malate shunt, which converts PEP to Pyr via three steps, phosphoenolpyruvate carboxykinase (Pepck; PEP + GDP + CO_2_→OAA + GTP), malate dehydrogenase (Mdh; OAA + NADH→Mal + NAD^+^), and malic enzyme (ME; Mal + NADP^+^→Pyr + CO_2_ + NADPH + H^+^) (18, 20, 21, 26) (Fig. 1). These unique aspects of glycolysis in *C. thermocellum*, particularly the use of PP_i_-Pfk and Ppdk, may result in greater energy yield (ATP or GTP) per glucose but also can be expected to significantly impact the thermodynamic driving force of individual reactions and of the overall pathway.

Recently developed experimental approaches for estimating *in vivo* Gibbs free energies (*ΔG*) of metabolic reactions have been applied to investigate the thermodynamics of glycolytic pathways in a few model organisms and biofuel producers (27–29). The estimation of *ΔG* by these approaches relies on the fundamental relation of *ΔG = −RT* ln(*J^+^/J^−^*) (30) and the determination of forward (*J^+^*) to backward (*J^−^*) flux ratios (*J^+^/J^−^*) from isotope tracer experiments (*R* is the gas constant and *T* is the temperature in kelvin) (27, 29). These studies, together with theoretical and computational advances, have provided new insights on the connection between pathway thermodynamics, flux, and enzyme efficiency (31–34).

Here, we integrated quantitative metabolomics with ^2^H and ^13^C metabolic flux analysis to investigate the *in vivo* reversibility and thermodynamics of the glycolytic pathways and central metabolic networks of *T. saccharolyticum* and *C. thermocellum*. We show that glycolysis in *C. thermocellum* is highly reversible at every step of the pathway and operates close to thermodynamic equilibrium. In comparison, the glycolytic pathway in *T. saccharolyticum* is highly thermodynamically favorable, comparable to that of anaerobically grown Escherichia coli. We also found that the ethanol fermentation pathway of *C. thermocellum* was substantially more reversible than that of *T. saccharolyticum*. Besides revealing differences in pathway reversibility, our analyses also represent the first experimental isotope-based reconstruction of the *T. saccharolyticum* central metabolic network. We found that this bacterium metabolizes glucose exclusively via the EMP pathway, while the Entner-Doudoroff (ED) pathway and the oxidative pentose phosphate pathway (oxPPP) are inactive. Our analyses also revealed a bifurcated tricarboxylic acid (TCA) cycle and a sedoheptulose bisphosphate bypass within the pentose phosphate pathway (PPP). Finally, we present evidence supporting the activity of several currently unannotated amino acid biosynthetic pathways in *T. saccharolyticum.*

## RESULTS

### Reconstruction of the *T. saccharolyticum* central metabolic network.

The application of ^2^H and ^13^C metabolic flux analysis to measure *in vivo* reversibility and thermodynamics of metabolic reactions requires prior knowledge of the underlying metabolic network topology. Recent studies have used isotope tracers, metabolic flux analysis (MFA), proteomics, or biochemical assays to elucidate the central metabolic network of *C. thermocellum* (14, 18, 20, 21, 24, 26, 35–38). However, although the genome sequence of *T. saccharolyticum* is available and systems-level approaches were used to characterize its physiology (22, 23, 39, 40), an isotope-based reconstruction of its metabolic network is currently lacking. To address this knowledge gap, we performed parallel steady-state labeling experiments by growing cells in [1-^13^C_1_]glucose, [6-^13^C_1_]glucose, [1,2-^13^C_2_]glucose, or a 50:50 mixture of [U-^13^C_6_]glucose and unlabeled glucose. Using these ^13^C labeling data, we first carried out a manual evaluation and reconstruction of the *T. saccharolyticum* central metabolic network. In a subsequent section (i.e., “^2^H and ^13^C metabolic flux analysis,” below), we present a quantitative MFA that corroborates the metabolic network reconstruction presented in this section.

### (i) Glucose catabolism.

**FIG 2:**
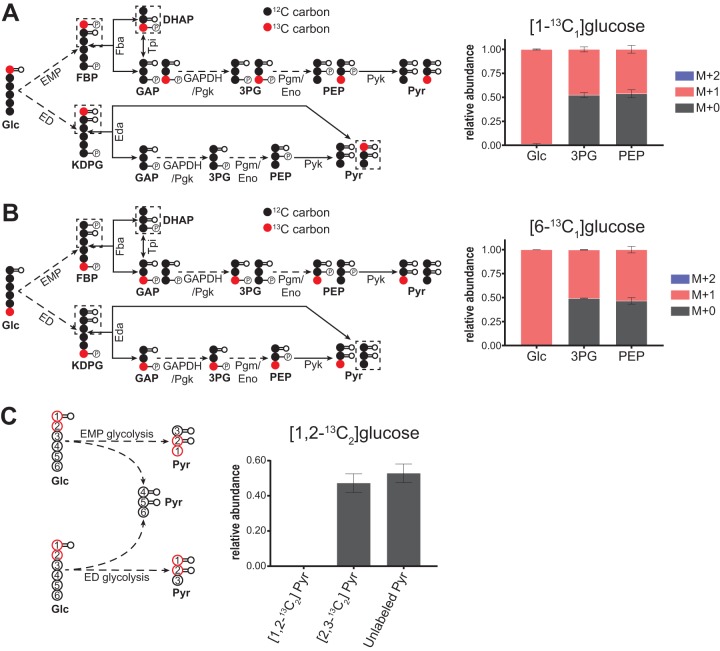
EMP is the primary glycolytic route in *T. saccharolyticum*. (A and B) Labeling of lower glycolytic intermediates 3PG and PEP when *T. saccharolyticum* was fed [1-^13^C_1_]glucose or [6-^13^C_1_]glucose. In both cases, 3PG and PEP were ∼50% M + 0 and ∼50% M + 1, which was consistent with their production exclusively via EMP glycolysis. The diagrams on the left depict predicted metabolite labeling via EMP or ED glycolysis. Solid arrows represent a single reaction; dashed arrows represent multiple reaction steps. ^13^C-labeled carbons are colored red; ^12^C-carbons are colored black. Data are averages from 2 independent biological replicates. Error bars show ± standard deviations (SD). (C) As shown in the diagram, glucose catabolism by the EMP and ED pathways results in different ordering of carbons in the pyruvate formed from the first three carbons of glucose. When *T. saccharolyticum* was fed [1,2-^13^C_2_]glucose, essentially all pyruvate was either unlabeled or labeled at the second (i.e., carbonyl group) and third (i.e., methyl group) carbons (i.e., [2,3-^13^C_2_]Pyr), consistent with exclusive use of the EMP pathway. If the ED pathway were active, pyruvate would have been labeled at the first and second carbons (i.e., [1,2-^13^C_2_]Pyr), but we detected <0.1% of this form of pyruvate. ^13^C-labeled carbons are represented as open circles with a red outline; ^12^C-labeled carbons are represented as open circles with a black outline. The number in each circle corresponds to their original position in glucose. Data are averages from 2 independent biological replicates. Error bars show ±SD. Abbreviations: ED, Entner-Doudoroff pathway; Eda, KDPG aldolase; KDPG, 2-keto-3-dehydro-6-phosphogluconate.

The Embden-Meyerhof-Parnas (EMP) and the Entner-Doudoroff (ED) pathways are the two most common glycolytic pathways found in microbial species, although several other variants have been described (20, 31, 41–43). The EMP and ED pathways share a common set of reactions that metabolize glyceraldehyde-3-phosphate (GAP) into pyruvate (Pyr) (i.e., lower glycolysis), but they differ in their initial steps (Fig. 2A and B). In the ED pathway, the intermediate 2-keto-3-deoxy-6-phosphogluconate (KDPG) is cleaved into Pyr (first three carbons) and GAP (last three carbons). Therefore, unlike in the EMP pathway (in which two GAP molecules are produced from glucose), the first three carbons of glucose bypass lower glycolysis in the ED pathway. Additionally, carbons 1, 2, and 3 of glucose catabolized via the ED pathway become the carboxyl, carbonyl, and methyl carbons, respectively, of Pyr, which is the reverse order of that obtained via the EMP pathway (Fig. 2C). *T. saccharolyticum* has genes encoding all of the enzymes in the EMP pathway, but its genome annotation does not support a complete ED pathway. *T. saccharolyticum* does not have sequences associated with the ED enzymes phosphogluconolactonase (Pgl; gluconolactone phosphate [GLP] + H_2_O→6-phosphogluconate [6PG]) or phosphogluconate dehydratase (Edd; 6PG→KDPG), although it does have genes annotated as glucose-6-phosphate dehydrogenase [G6pdh; G6P + NAD(P)^+^→GLP + NAD(P)H, *Tsac_1610*] and ED pathway aldolase (Eda; KDPG→GAP + Pyr; *Tsac_0268*) (22, 23).

When *T. saccharolyticum* was grown on [1-^13^C_1_]- or [6-^13^C_1_]glucose, the glycolytic intermediates 3-phosphoglycerate (3PG) and phosphoenolpyruvate (PEP) both were ∼50% M + 0 (i.e., unlabeled; all carbons are ^12^C, where M represents the unlabeled parent mass) and ∼50% M + 1 (i.e., containing one ^13^C carbon), which was consistent with their production via EMP glycolysis (Fig. 2A and B). If the ED pathway was the exclusive glycolytic pathway, 3PG and PEP would have been 100% M + 0 in [1-^13^C_1_]glucose or 100% M + 1 in [6-^13^C_1_]glucose (27). If both the EMP and ED pathways were active, labeling of 3PG and PEP would have been <50% M + 1 in [1-^13^C_1_]glucose and >50% M + 1 in [6-^13^C_1_]glucose. For example, equal utilization of the EMP and ED pathways would result in 25% M + 1 3PG and PEP in [1-^13^C_1_]glucose and 75% M + 1 3PG and PEP in [6-^13^C_1_]glucose. As additional support for the absence of ED pathway activity, when *T. saccharolyticum* was fed [1,2-^13^C_2_]glucose, Pyr was labeled only at the second and third carbon (i.e., [2,3-^13^C_2_]Pyr), consistent with its production solely via the EMP pathway (Fig. 2C). We did not detect Pyr labeled at the first and second carbons (i.e., [1,2-^13^C_2_]Pyr), which would have been indicative of ED pathway activity (27).

Finally, as shown in Table S1 in the supplemental material, high intracellular levels of the EMP intermediates FBP and dihydroxyactone phosphate (DHAP), which were comparable to those in anaerobic E. coli, a known EMP-utilizing organism, together with nondetectable levels of the ED pathway intermediates 6PG and KDPG, which are present in the ED pathway, utilizing organisms such as Z. mobilis, was consistent with the existence of an active EMP pathway and an inactive ED pathway in *T. saccharolyticum* (27, 44).

10.1128/mSystems.00736-19.5 Table S1 Absolute intracellular metabolite concentrations in *C. thermocellum*, *T. saccharolyticum*, and anaerobically grown E. coli. Download Table S1, XLSX file, 0.01 MB. Copyright © 2020 Jacobson et al. 2020 Jacobson et al. This content is distributed under the terms of the Creative Commons Attribution 4.0 International license.

### (ii) Pentose phosphate pathway and sedoheptulose bisphosphate bypass.

**FIG 3:**
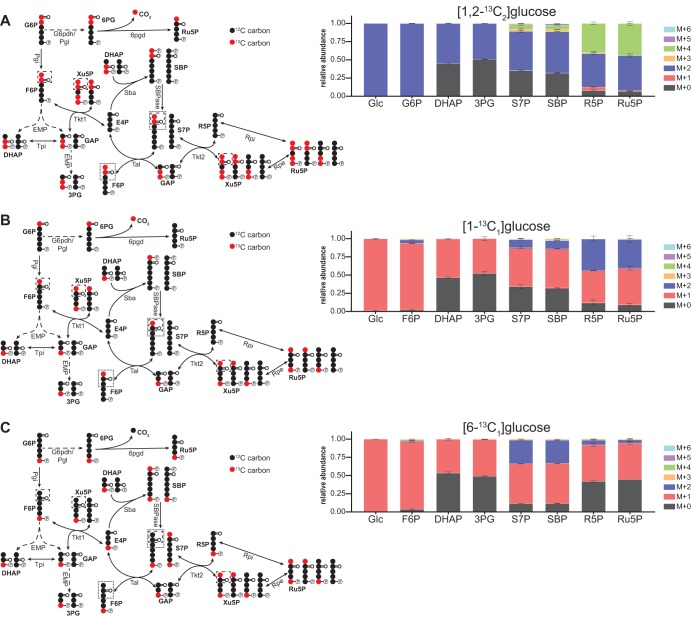
*T. saccharolyticum* has two nonoxidative routes for pentose phosphate production. Labeling of glycolytic and PPP intermediates when *T. saccharolyticum* was fed [1,2-^13^C_2_]glucose (A), [1-^13^C_1_]glucose (B), or [6-^13^C_1_]glucose (C). As described in the text, labeling patterns indicated that the oxidative branch of the PPP was essentially inactive and suggested the presence of a sedoheptulose bisphosphate bypass, comprising SBP aldolase (Sba) and SBP phosphatase (SBPase). The diagrams on the left show predicted metabolite labeling with the specified isotope tracer. Solid arrows represent a single reaction; dashed arrows represent multiple reaction steps. Dotted boxes indicate carbons transferred during transaldolase and transketolase reactions. ^13^C-labeled carbons are colored red; ^12^C-labeled carbons are colored black. Data are averages from 2 or 3 independent biological replicates. Error bars show ±SD. Abbreviations: 6PG, 6-phosphogluconate; 6pgd, 6-phosphogluconate dehydrogenase; E4P, erythrose-4-phosphate; G6pdh, glucose-6-phosphate dehydrogenase; R5P, ribose-5-phosphate; Rpe, ribose phosphate epimerase; Rpi, ribose phosphate isomerase; Ru5P, ribulose-5-phosphate; S7P, sedoheptulose-7-phosphate; Sba, sedoheptulose-1,7-bisphosphate aldolase; SBP, sedoheptulose-1,7-bisphosphate; SBPase, sedoheptulose-1,7-bisphosphate phosphatase; Tal, transaldolase; Tkt1, transketolase reaction 1; Tkt2, transketolase reaction 2; Xu5P, xylulose-5-phosphate.

The oxPPP converts glucose-6-phosphate (G6P) to 6PG via the G6pdh and Pgl reactions, which are shared with the ED pathway. 6PG then is decarboxylated by 6-phosphogluconate dehydrogenase [6pgd; 6PG + NAD(P)^+^→Ru5P + CO_2_ + NAD(P)H] to form ribulose-5-phosphate (Ru5P), losing what was originally the first carbon of glucose as CO_2_ (Fig. 3). *T. saccharolyticum* has annotated genes for *g6pdh* (*Tsac_1610*) and *6pgd* (*Tsac_0154*) genes, but there are no annotated *pgl* sequences (22, 23, 45). Our labeling data indicated that the oxPPP was essentially inactive in *T. saccharolyticum*, since less than 1% of Ru5P was present as M + 1 when cells were grown on [1,2-^13^C_2_]glucose (Fig. 3A). Nondetectable levels of the intermediate 6PG were consistent with the lack of an active oxPPP (46, 47).

The nonoxidative production of pentose phosphates typically proceeds via a transketolase reaction (Tkt1; F6P + GAP→E4P + Xu5P), a transaldolase reaction (Tal; E4P + F6P→S7P + GAP), and a second transketolase reaction (Tkt2; S7P + GAP→R5P + Xu5P). Additionally, ribose phosphate epimerase (Rpe; Xu5P→Ru5P) and ribose phosphate isomerase (Rpi; Ru5P→R5P) interconvert Xu5P, Ru5P, and R5P. In *T. saccharolyticum*, R5P and Ru5P were predominantly M + 2 and M + 4 labeled in [1,2-^13^C_2_]glucose and M + 1 and M + 2 labeled in [1-^13^C_1_]glucose (Fig. 3B and C), which was consistent with their production via the nonoxidative PPP reactions listed above.

Interestingly, we found the production of a large fraction (30 to 40%) of M + 0 S7P in both [1,2-^13^C_2_]- and [1-^13^C_1_]glucose that could not be explained by canonical nonoxidative PPP reactions (Fig. 3A and B). For example, taking into account the observed labeling patterns of PPP intermediates during growth in [1,2-^13^C_2_]- or [1-^13^C_1_]glucose, no more than 10% M + 0 S7P should be present under either condition if Tal and Tkt1 were the only reactions producing S7P. Therefore, the large unexpected fraction of M + 0 S7P suggested that another route for S7P production should be present. Specifically, these data suggested that *T. saccharolyticum* possesses a sedoheptulose bisphosphate (SBP) bypass comprising an SBP aldolase (Sba; DHAP + E4P→SBP) and an SBP phosphatase (SBPase; SBP + P_i_→S7P + pyrophosphate [PP_i_]) (24, 26). While there are no genes associated with SBPase activity in *T. saccharolyticum*, several genes (i.e., *Tsac_0260*, *Tsac_0328*, and *Tsac_2313*) are annotated as both Fbas and Sbas (22, 23), and one or more of these could be responsible for the observed Sba activity (48).

This SBP bypass can explain the production of unlabeled S7P from unlabeled DHAP and E4P during growth on [1-^13^C_1_]- and [1,2-^13^C_2_]glucose. Similarly, during [6-^13^C_1_]glucose labeling, the large fraction of M + 2 S7P (∼32.5%) was also indicative of SBP bypass activity (Fig. 3C). Alternative PPP routes, such as phosphoketolase, formaldehyde transketolase, and the reverse ribulose monophosphate (RuMP) pathway, cannot explain the observed PPP labeling patterns (Fig. S1).

10.1128/mSystems.00736-19.1 FIG S1 Alternative routes for pentose phosphate generation and bifurcation of the TCA cycle in *T. saccharolyticum.* (A) [1,2-^13^C_2_]glucose labeling indicated that formaldehyde transketolase (Ftkt) was unlikely responsible for the formation of Ru5P and R5P. Dihydroxyacetone phosphate (DHAP) was either unlabeled or M + 2 labeled and can be dephosphorylated to form dihydroxyacetone (DHA). Ftkt condenses the first two carbons of DHA with glyceraldehyde-3-phosphate (GAP, which was inferred to be also M + 0 or M + 2) to form xylulose-5-phosphate (Xu5P), releasing the third carbon of DHA as formaldehyde. Because DHA is symmetrical, carbons 1 and 3 are equivalent, so a ^13^C carbon from M + 2 DHA has a 50% chance of being incorporated into Xu5P. Thus, Xu5P produced by Ftkt inherits 0 or 2 ^13^C-labeled carbons from GAP and 0, 1, or 2 ^13^C carbons from DHA. Xu5P is epimerized to form ribulose-5-phosphate (Ru5P), which is isomerized to form ribose-5-phosphate (R5P). Therefore, if Ftkt was involved in the production of pentose phosphates, we would expect Xu5P, Ru5P, and R5P to be approximately 17% M + 0, 17% M + 1, 33% M + 2, 17% M + 3, and 17% M + 4. However, labeling of Ru5P and R5P in cells fed [1,2-^13^C_2_]glucose did not match these patterns but were primarily M + 2 and M + 4 labeled, with only small fractions of M + 1 or M + 3. These labeling patterns are better explained by canonical nonoxidative PPP reactions supplemented with an SBP bypass (Fig. 3). This agrees with the lack of an annotated gene encoding Ftkt in *T. saccharolyticum.* (B) [1,2-^13^C_2_]glucose labeling also indicated that the reverse ribulose monophosphate (rRuMP) pathway was not responsible for the formation of Ru5P and R5P. The rRuMP pathway isomerizes fructose-6-phosphate (F6P) to hexulose-6-phosphate (Hu6P). Hu6P is split into (Ru5P) and formaldehyde, with carbons 2 to 6 retained in Ru5P. Thus, the carbon lost as formaldehyde is ^13^C labeled during [1,2-^13^C_2_]glucose labeling, leading to the production of M + 1 Ru5P. Ru5P then is isomerized to form R5P. Ru5P and R5P were primarily M + 2 and M + 4 during [1,2-^13^C_2_]glucose labeling, with only a very small M + 1 fraction, indicating that pentose phosphate generation via the rRuMP pathway was unlikely. This agrees with the lack of an annotated gene encoding hexulose-6-phosphate synthase (Hps) in *T. saccharolyticum.* (C) [6-^13^C_1_]glucose labeling indicated that phosphoketolase (Pkt) activity was unlikely to be responsible for the formation of Ru5P and R5P. GAP produced by the EMP pathway was M + 0 or M + 1. GAP is further processed to form acetyl-phosphate (AcP), which was M + 0 or M + 1 labeled. GAP and AcP can be condensed by Pkt to form Xu5P, which thus inherits no or one ^13^C-labeled carbon from GAP and 0 or 1 ^13^C-labeled carbon from AcP. Thus, Xu5P, Ru5P, and R5P produced via Pkt were expected to be ∼25% M + 0, ∼50% M + 1, and ∼25% M + 2. However, we observed approximately equal proportions of M + 0 and M + 1 Ru5P and R5P, with only a small fraction of M + 2, indicating that Pkt was unlikely to be responsible for pentose phosphate generation in *T. saccharolyticum*. This agrees with the lack of an annotated gene encoding a phosphoketolase in *T. saccharolyticum.* (D) Labeling of TCA cycle intermediates in *T. saccharolyticum* fed [1-^13^C_1_]glucose. M + 2 AKG production was consistent with production from oxaloacetate and acetyl-CoA via the oxidative TCA cycle. A lack of M + 2 succinate indicates that succinate was not produced oxidatively from AKG and that the TCA cycle was bifurcated. The presence of only M + 0 and M + 1 succinate, malate, and aspartate indicates these metabolites were produced by a reductive TCA. (E) Labeling of TCA cycle intermediates in *T. saccharolyticum* fed [1,2-^13^C_2_]glucose. M + 4 AKG production was consistent with production from oxaloacetate and acetyl-CoA via the oxidative TCA cycle. A lack of M + 3 succinate indicates that succinate was not produced oxidatively from AKG and that the TCA cycle was bifurcated. The presence of only M + 0 and M + 2 succinate, malate, and aspartate indicates these metabolites were produced by a reductive TCA. The increased proportion of M + 0 α-ketoglutarate, pyruvate, succinate, and malate were due to variable extracellular pools of these metabolites that do not participate in metabolism. In addition to its production via Fumh, fumarate may be produced by deamination of aspartate, which takes place in a number of biosynthetic pathways, such as purine biosynthesis. This route of fumarate production would result in the same labeling patterns as its production from oxaloacetate via Mdh and Fumh. CO_2_ labeling was inferred from the MIDs of citrulline and ornithine, as described in Materials and Methods. The diagrams on the left show predicted metabolite labeling with the specified isotope tracer. Pyruvate formate lyase (Pfl) was omitted from panels D and E for clarity but serves the same role as Pfor. The diagrams on the left show predicted metabolite labeling with the specified isotope tracer. Solid arrows represent a single reaction; dashed arrows represent multiple reaction steps. ^13^C-labeled carbons are colored red; ^12^C carbons are colored black. Carbons with an approximately equal chance of being ^13^C or ^12^C labeled are represented by a half red and half black circle. Data are averages from 2 to 3 independent biological replicates. Error bars show ±SD. Abbreviations: Dap, dihydroxyacetone phosphate phosphorylase; DHA, dihydroxyacetone; FAL, formaldehyde; Ftkt, formaldehyde transketolase; Hps, hexulose-6-phosphate synthase; Hu6P, hexulose-6-phosphate; Phi, phosphohexuloisomerase; Pkt, phosphoketolase. Download FIG S1, PDF file, 0.6 MB. Copyright © 2020 Jacobson et al. 2020 Jacobson et al. This content is distributed under the terms of the Creative Commons Attribution 4.0 International license.

### (iii) TCA cycle.

**FIG 4:**
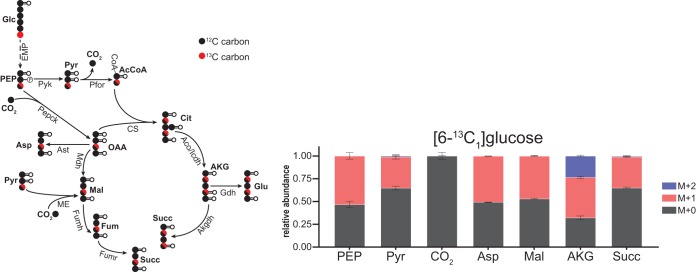
*T. saccharolyticum* TCA cycle is bifurcated. Labeling of lower glycolytic and TCA cycle intermediates when *T. saccharolyticum* was fed [6-^13^C_1_]-glucose. α-Ketoglutarate (AKG) labeling patterns were consistent with its production from oxaloacetate and acetyl-CoA via an oxidative TCA. The presence of M + 2 α-ketoglutarate but near absence of M + 2 succinate indicated that the TCA cycle was bifurcated. Labeling patterns in succinate, malate, and aspartate (used as a surrogate for oxaloacetate) were consistent with their production via a reductive TCA. Experiments with [1-^13^C_1_] and [1,2-^13^C_2_]glucose confirmed these findings (Fig. S2). In addition to its production via Fumh, fumarate can be produced by deamination of aspartate, which occurs in biosynthetic pathways such as purine biosynthesis. This route of fumarate production would result in the same labeling patterns as its production from oxaloacetate via Mdh and Fumh. The increased proportion of M + 0 pyruvate, succinate, α-ketoglutarate, and malate was due to extracellular pools of these metabolites that do not become labeled. CO_2_ labeling was inferred from the mass isotopomer distributions (MIDs) of citrulline and ornithine, as described in Materials and Methods. The diagram on the left shows predicted metabolite labeling with [6-^13^C_1_]glucose. Pyruvate formate lyase (Pfl) was omitted from the figure but serves the same role as Pfor. Solid arrows represent a single reaction; dashed arrows represent multiple reaction steps. ^13^C-labeled carbons are colored red; ^12^C-labeled carbons are colored black. Data are averages from 2 independent biological replicates. Error bars show ±SD. Abbreviations: AcCoA, acetyl-coenzyme A; Aco, aconitase; AKG, α-ketoglutarate; Akgdh, α-ketoglutarate dehydrogenase; Asp, aspartate; Ast, aspartate transaminase; Cit, citrate; CoA, coenzyme A; CS, citrate synthase; Fum, fumarate; Fumh, fumarate hydratase; Fumr, fumarate reductase; Gdh, glutamate dehydrogenase; Glu, glutamate; Icdh, isocitrate dehydrogenase; Pfor, pyruvate ferredoxin oxidoreductase; Succ, succinate.

Several TCA cycle enzymes remain unannotated in the *T. saccharolyticum* genome, including citrate synthase and malate dehydrogenase. Our ^13^C tracer data indicated that the TCA cycle in *T. saccharolyticum* functions as a branched pathway to produce essential biosynthetic precursors: α-ketoglutarate (AKG) was produced oxidatively despite the lack of an annotated citrate synthase, while oxaloacetate (OAA), malate (Mal), and succinate all were produced reductively. During growth in [6-^13^C_1_]glucose, α-ketoglutarate (AKG) labeling patterns were consistent with its production from oxaloacetate and acetyl-coenzyme A (AcCoA) via citrate and isocitrate (Fig. 4). However, although AKG was ∼23% M + 2, succinate was <0.6% M + 2, indicating negligible oxidative succinate production from AKG via an AKG dehydrogenase. Although several genes in *T. saccharolyticum* are annotated as a 2-oxoacid/AKG:ferredoxin dehydrogenase, there is no gene specifically annotated as an AKG dehydrogenase (22, 23, 45). Instead, labeling patterns in succinate, Mal, and aspartate (here being used as a surrogate for OAA) were consistent with their production via a reductive TCA (Fig. 4). As detailed in Fig. S1, [1-^13^C_1_]- and [1,2-^13^C_2_]glucose labeling confirmed the production of succinate, Mal, and aspartate via the reductive TCA.

10.1128/mSystems.00736-19.2 FIG S2 Routes for amino acid synthesis. When the EMP pathway is used for glucose catabolism, TCA cycle intermediates have equivalent labeling during [1-^13^C_1_]- and [6-^13^C_1_]glucose labeling. Thus, a single diagram was used to represent labeling with both [1-^13^C_1_]- and [6-^13^C_1_]glucose in panels A and B. (A) Labeling of aspartate, asparagine, and threonine was consistent with all three amino acids originating from oxaloacetate (OAA). OAA can be produced via carboxylation (CO_2_ addition) of PEP by Pepck or via carboxylation of Pyr by ME to produce malate, followed by its conversion to OAA by Mdh. Because PEP and Pyr were both ∼50% M + 0 and ∼50% M + 1 and CO_2_ was unlabeled, production of ∼50% M + 0 and ∼50% M + 1 OAA was expected. Aspartate, produced via amination of OAA, had the same labeling pattern as Mal, PEP, and Pyr (∼50% M + 0 and ∼50% M + 1). Aspartate is further converted to threonine (Thr) or asparagine (Asn) without carbon transitions, resulting in Asn and Thr labeling that matched that in PEP and Pyr (∼50% M + 0 and ∼50% M + 1). (B) Labeling of amino acids derived from α-ketoglutarate (AKG) was consistent with canonical synthesis pathways. OAA is formed by the addition of CO_2_ to PEP by Pepck or to Pyr by the combined action of ME and Mdh. Because PEP and Pyr are both ∼50% M + 0 and ∼50% M + 1 and CO_2_ is unlabeled, M + 0 and M + 1 OAA are produced. Pyr is also decarboxylated by Pfor, losing an unlabeled CO_2_ to form acetyl-CoA (AcCoA) that is ∼50% M + 0 and ∼50% M + 1. Citrate synthase combines OAA with AcCoA to form citrate, which is ∼25% M + 0, ∼50% M + 1, and ∼25% M + 2, and has a 50% chance of inheriting one ^13^C carbon each from AcCoA and OAA. Citrate then is decarboxylated to form AKG, losing an unlabeled carbon in the process, leaving AKG with the same labeling pattern as citrate (∼25% M + 0, ∼50% M + 1, and ∼25% M + 2). AKG is aminated to form glutamate, which serves as the precursor to glutamine, proline, ornithine, and citrulline, all of which are approximately ∼25% M + 0, ∼50% M + 1, and ∼25% M + 2 labeled, consistent with the formation of all of these amino acids from AKG. (C) Labeling of *T. saccharolyticum* with [1,2-^13^C_2_]glucose indicated that valine and leucine were produced via canonical synthesis pathways from pyruvate and acetyl-CoA. Briefly, two pyruvate molecules are condensed to form the intermediate ketoisovalerate (KIV), losing the carboxyl group of one of the pyruvate molecules as CO_2_. Each pyruvate has a ∼50% chance of being labeled at the carbonyl and methyl groups, meaning KIV should be primarily ∼25% M + 0, ∼50% M + 2, and ∼25% M + 4. KIV is aminated to form valine, and we detect valine labeling that approximates what we expect from KIV. KIV is also used for leucine biosynthesis, where it receives an additional two carbons from AcCoA, itself formed from the carbonyl and methyl carbons of pyruvate and, thus, ∼50% M + 0 and ∼50% M + 2. During leucine synthesis, the carboxyl carbon of KIV (originally a carboxyl carbon of pyruvate) is lost as CO_2_. Thus, leucine independently inherits 3 sets of 2 carbons, and each set may be both labeled or unlabeled and should be labeled ∼12.5% M + 0, ∼37.5% M + 2, ∼37.5% M + 4, and ∼12.5% M + 6. As expected, leucine labeling is consistent with production from KIV, although it is enriched for the M + 0-labeled form due to incomplete label penetration. The presence of only M + 0 and M + 2 labeling in acetyl group carbons confirms the EMP pathway as the sole glycolytic route, as ED pathway activity would lead to the formation of M + 1 acetyl groups via decarboxylation of pyruvate due to the alternate orientation of carbons in pyruvate formed from the first three carbons of glucose via the ED pathway. (D) [1-^13^C_1_]glucose labeling in *T. saccharolyticum* confirmed the synthesis of aromatic amino acids via the shikimate pathway. Erythrose-4-phosphate (E4P) is produced by transketolase from the bottom 4 carbons of F6P. The reverse activity of the Pfk and Fba reactions in the EMP pathway can result in F6P that is labeled at the 6th carbon. Thus, E4P can be M + 0 or M + 1, although the M + 0 form is much more prevalent. The shikimate pathway condenses E4P with PEP (also M + 0 or M + 1 due to formation via the EMP pathway) to form a seven-carbon intermediate, 3-deoxy-d-arabinoheptulosonate-7-phosphate (DAHP), which is processed to become shikimate-3-phosphate (Sh3P). Sh3P can contain 0, 1, or 2 labeled carbons. Sh3P is further processed and combined with another PEP molecule (which is M + 0 or M + 1) and dephosphorylated to form chorismate, which is M + 0, M + 1, M + 2, or M + 3. Chorismate mutase converts chorismate to prephenate, which is decarboxylated, dehydrated, and aminated to form phenylalanine (Phe) or decarboxylated, oxidized, and aminated to form tyrosine (Tyr). The CO_2_ lost by decarboxylation is unlabeled (originally the first carbon of PEP), meaning the labeling of Phe and Tyr is the same as that of chorismate. Therefore, Phe and Tyr should inherit up to one ^13^C carbon from E4P and up to one ^13^C carbon from each of the two PEPs, resulting in M + 0, M + 1, M + 2, or M + 3 Phe and Tyr. The experimental labeling patterns in Phe and Tyr matched these predictions. Pyruvate formate lyase (Pfl) was omitted from panels A and B for clarity but serves the same role as Pfor. The increased proportion of M + 0 α-ketoglutarate and malate in panels A and B was due to extracellular pools of these metabolites that do not become labeled. The diagrams on the left show predicted metabolite labeling with the specified isotope tracer. Solid arrows represent a single reaction; dashed arrows represent multiple reaction steps. ^13^C-labeled carbons are colored red; ^12^C carbons are colored black. Carbons with an approximately equal chance of being ^13^C or ^12^C labeled are represented by a half red and half black circle. Data are averages from 2 to 3 independent biological replicates. Error bars show ±SD. Abbreviations: Asn, asparagine; Citr, citrulline; Gln, glutamine; Leu, leucine; Orn, ornithine; Phe, phenylalanine; Sh3P, shikimate-3-phosphate; Tyr, tyrosine; Val, valine. Download FIG S2, PDF file, 0.6 MB. Copyright © 2020 Jacobson et al. 2020 Jacobson et al. This content is distributed under the terms of the Creative Commons Attribution 4.0 International license.

### (iv) TCA cycle anaplerosis.

**FIG 5:**
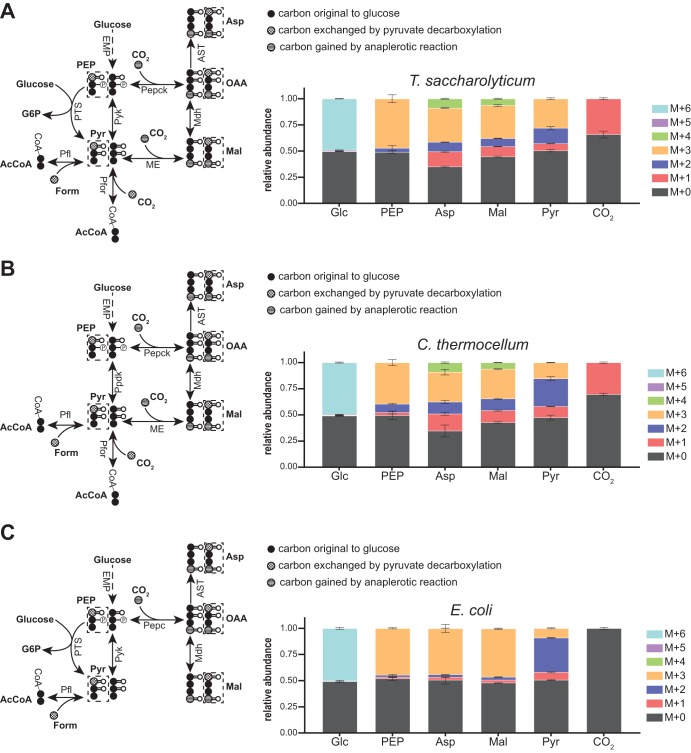
Reversibility of PEP-to-Pyr conversion. Steady-state labeling patterns of lower glycolytic intermediates in *T. saccharolyticum* (A), *C. thermocellum* (B), and E. coli (C) during anaerobic growth on an equimolar mixture of [U^13^C_6_]glucose and unlabeled glucose. In all three bacteria, forward glycolytic flux produced M + 0 or M + 3 PEP and pyruvate. Carboxylation of PEP or pyruvate to form oxaloacetate (OAA) or malate (Mal) resulted in M + 4 OAA/Mal (from M + 3 pyruvate and labeled CO_2_) or M + 1 OAA/Mal (from M + 0 pyruvate and labeled CO_2_). Reversible decarboxylation of pyruvate by Pfor or Pfl resulted in the production of M + 1 pyruvate (from M + 0 pyruvate and labeled CO_2_/formate) and M + 2 pyruvate (from M + 3 pyruvate and unlabeled CO_2_/formate). Once formed, M + 1 or M + 2 pyruvate propagated back to PEP. The higher proportion of M + 1 or M + 2 PEP in *C. thermocellum* indicated that the conversion of PEP to pyruvate was most reversible in this bacterium. *T. saccharolyticum* displayed only moderate reversibility of this conversion, while E. coli displayed almost no reversibility. CO_2_ labeling was inferred from the MIDs of citrulline and ornithine, as described in Materials and Methods. The pyruvate MID was inferred from the MID of valine. The diagrams on the left display the reactions surrounding the PEP/pyruvate node of each bacteria. Black circles represent carbons that originate in glucose; dotted circles represent carbons that can be exchanged by the pyruvate decarboxylating reactions (Pfor or Pfl); striped circles represent carbons that are gained during the anaplerotic reactions (ME, Pepck, and Pepc). Solid arrows represent a single reaction; dashed arrows represent multiple reaction steps. Data are averages from 2 independent biological replicates. Error bars show ±SD. Abbreviations: Form, formate; Pepc, phosphoenolpyruvate carboxylase; Pfl, pyruvate formate lyase; PTS, phosphotransferase system.

Anaplerotic reactions transform PEP and Pyr into TCA cycle intermediates, such as OAA and Mal. According to its genome annotation, *T. saccharolyticum* has a malic enzyme (ME; Mal + NADP^+^→Pyr + CO_2_ + NADPH + H^+^; *Tsac_0488*, *Tsac_0975*) and a phosphoenolpyruvate carboxykinase (Pepck; OAA + ATP→PEP + CO_2_ + ADP; *Tsac_2170*), which is typically considered a gluconeogenic enzyme (22, 23, 45). When *T. saccharolyticum* was fed an equimolar mixture of [U-^13^C_6_]glucose and unlabeled glucose, some M + 2 pyruvate (14.7%) was produced due to reversibility of pyruvate ferredoxin oxidoreductase (Pfor; Pyr + CoA + Fd_ox_ → AcCoA + CO_2_ + Fd_red_) or pyruvate formate lyase (Pfl; Pyr + CoA → AcCoA + Form) (M + 1 pyruvate is produced in a similar fashion). Thus, the presence of M + 2 aspartate (surrogate for OAA) and M + 2 Mal indicated production of these two metabolites from pyruvate, likely via ME (Fig. 5A). However, the M + 2 fraction in aspartate (9.1%) and Mal (7.7%) was smaller than that of Pyr (*P* < 0.05 for both pairwise comparisons), which suggested that PEP, with an M + 2 fraction (3.9%) smaller than that of Pyr (*P* < 0*.05*), also contributed to the production of these two metabolites via Pepck or via an unannotated PEP carboxylase.

### (v) Amino acid synthesis and one-carbon metabolism.

Our ^13^C-tracer data confirmed that aspartate, asparagine, and threonine were produced via canonical routes starting from oxaloacetate (Fig. S2A). Similarly, our data supported production of glutamate, glutamine, ornithine, citrulline, and proline via canonical pathways starting with AKG (Fig. S2B). The amino acids valine and leucine were also produced via canonical branched-chain amino acid synthesis pathways from the intermediate ketoisovalerate (Fig. S2C). Finally, ^13^C-labeling patterns in the aromatic amino acids phenylalanine and tyrosine were consistent with their production via the shikimate pathway (Fig. S2D).

**FIG 6:**
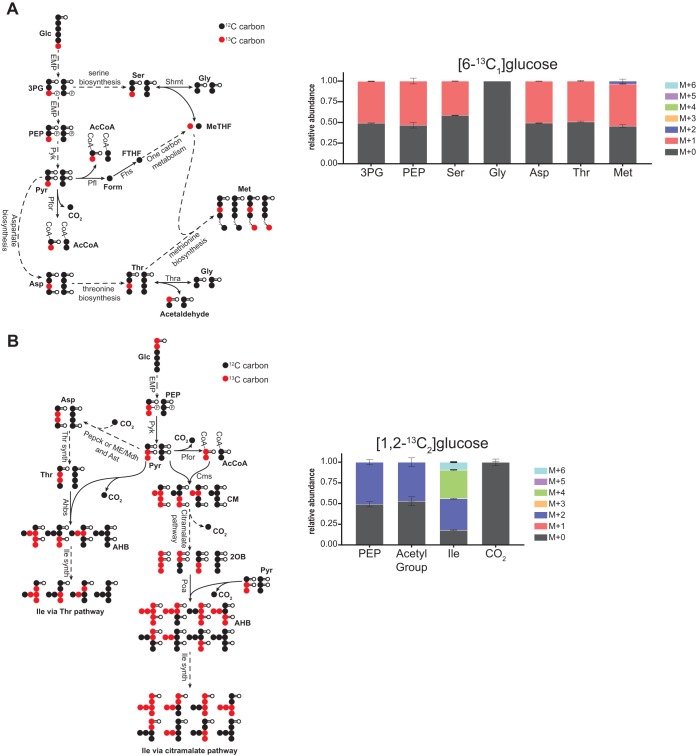
One carbon metabolism and aromatic amino acid production. (A) [6-^13^C_1_]glucose labeling indicated that in *T. saccharolyticum* serine was produced from both 3PG and glycine. In addition, the production of a small fraction of M + 2 methionine indicated the presence of ^13^C-labeled C1 units in 5-methyltetrahydrofolate (MTHF), which is produced from 5,10-methylenetetrahydrofolate (MeTHF). This implied partial production of C1 units from serine via SMHT. These conclusions were supported by [1-^13^C_1_] and [1,2-^13^C_2_]glucose labeling data (Fig. S3). (B) Isoleucine may be produced from threonine or via the citramalate pathway. [1,2-^13^C_2_]glucose labeling data were consistent with production of isoleucine via the citramalate pathway in *T. saccharolyticum*. Specifically, production of M + 6 isoleucine can only be explained by citramalate pathway activity, and the close-to-equal proportions of M + 0 to M + 6 and M + 2 to M + 4 labeled forms also support isoleucine production via this pathway. Labeling experiments using an equimolar mixture of [U-^13^C_6_]glucose and [U-^12^C_6_]glucose supported this conclusion (Fig. S3D). CO_2_ labeling was inferred from the MIDs of citrulline and ornithine. The diagrams on the left show predicted metabolite labeling with the specified isotope tracer. Solid arrows represent a single reaction; dashed arrows represent multiple reaction steps. ^13^C-labeled carbons are colored red; ^12^C-labeled carbons are colored black. Data are averages from 2 or 3 independent biological replicates. Error bars show ±SD. Pyruvate formate lyase (Pfl) was omitted from panels A and B for clarity but serve the same role as Pfor. Abbreviations: 2OB, 2-oxobutanoate; AHB, acetohydroxybutanoate; Ahbs, acetohydroxybutanoate synthase; CM, citramalate; Cms, citramalate synthase; Fhs, formate tetrahydrofolate ligase; FTHF, 10-formyltetrahydrofolate; Gly, glycine; Ile, isoleucine; KIV, keto-isovalerate; Met, methionine; MeTHF, 5,10-methylenetetrahydrofolate; Poa, pyruvate oxobutanoate acetaldehyde transferase; Ser, serine; Shmt, serine hydroxymethyltransferase; THF, tetrahydrofolate; Thr, threonine; Thra, threonine aldolase.

Although the genes encoding the enzymes responsible for producing serine from 3PG are not annotated in *T. saccharolyticum*, our data indicated that serine was produced primarily from 3PG (Fig. 6A and Fig. S3A and B). However, serine can also be synthesized from glycine via serine hydroxymethyltransferase (Shmt; 5,10-methylenetetrahydrofolate [MeTHF] + glycine→serine + tetrahydrofolate; *Tsac_1185* [22, 23]), and our data suggested that a measurable portion of the serine pool (i.e., ∼22%) was derived (either net production or exchange) from glycine via Shmt (Fig. 6A and Fig. S3A and B).

10.1128/mSystems.00736-19.3 FIG S3 One-carbon metabolism, purine biosynthesis, and the citramalate pathway. (A) Labeling of serine when *T. saccharolyticum* was fed [1-^13^C_1_]glucose confirmed production of serine from both 3PG and glycine. Serine was 56.0% M + 0, while 3PG was 52.3% M + 0 and glycine was 99.8% M + 0, indicating serine production from both 3PG and glycine. Labeling of methionine (5.5% M + 2) while threonine was only M + 0 and M + 1 confirms that the C1 unit in 5,10-methylenetetrahydrofolate (MeTHF) was labeled due to Shmt activity. (B) Labeling of serine when *T. saccharolyticum* was fed [1,2-^13^C_2_]glucose confirmed production of serine from both 3PG and glycine. Serine was 52.3% M + 0, while 3PG was 50.7% M + 0 and glycine was 53.1% M + 0. Additionally, serine was 42.3% M + 2 while 3PG was 50.8% M + 2 and glycine was 1.0% M + 2. Together, these data indicate serine production from both 3PG and glycine. Labeling of methionine (1.5% M + 3) while threonine was only M + 0, M + 1, and M + 2 confirms that the C1 unit in 5,10-methylenetetrahydrofolate (MeTHF) was labeled due to Shmt activity. (C) Labeling of purine nucleotides served as additional confirmation that C1 units in one carbon metabolism were ^3^C labeled during growth on [6-^13^C_1_]glucose. Purines are synthesized *de novo* via a series of reactions that incorporate ribose-5-phosphate (R5P), glycine (Gly), CO_2_, and two C1 units from 10-formyltetrahydrofolate (FTHF). During [6-^13^C_1_]glucose labeling in *T. saccharolyticum*, R5P is at most M + 2 labeled and CO_2_ and Gly are M + 0. The increased proportion of the M + 2 and M + 3 forms of ATP and GTP relative to R5P was due to the addition of labeled C1 units from FTHF during *de novo* purine biosynthesis. We used the MIDs of threonine and methionine to infer labeling of C1 units in MeTHF (represented in the figure as THF carbon). MeTHF is oxidized by one carbon metabolic reactions to form 5,10-methenyl-THF, which is dehydrated to form FTHF. We combined the inferred MeTHF labeling pattern with the labeling in R5P, Gly, and CO_2_ to produce an expected labeling pattern of a purine molecule synthesized from these precursors (represented on the figure as predicted purine), resulting in a labeling pattern that matched that of measured purines ATP and GTP. The match between the predicted purine labeling pattern and the measured pattern indicated that the labeling in MeTHF was able to substitute for that of FTHF, indicating the two metabolites are close to isotopic equilibrium. (D) Labeling with an equimolar mixture of [U-^13^C_6_] and [U-^12^C_6_]glucose confirmed the activity of the citramalate pathway. Production of isoleucine from threonine during labeling with an equimolar mixture of [U-^13^C_6_] and [U-^12^C_6_]glucose would result in 25% M + 3 isoleucine, but we observe only 1.5% M + 3 isoleucine, suggesting at most 6% contribution of the pathway starting from threonine. The diagrams on the left show predicted metabolite labeling with the specified isotope tracer. Solid arrows represent a single reaction; dashed arrows represent multiple reaction steps. ^13^C-labeled carbons are colored red; ^12^C carbons are colored black. Data are averages from 2 to 3 independent biological replicates. Error bars show ±SD. Download FIG S3, PDF file, 0.7 MB. Copyright © 2020 Jacobson et al. 2020 Jacobson et al. This content is distributed under the terms of the Creative Commons Attribution 4.0 International license.

Glycine was unlabeled during growth on [6-^13^C_1_]glucose and [1-^13^C_1_]glucose, consistent with its production either via threonine aldolase (Thra; threonine→glycine + acetaldehyde; *Tsac_0783* [22, 23]) or reverse Shmt (Fig. 6A and Fig. S3A). However, the production of a small fraction (3.6% during [6-^13^C_1_]glucose labeling) of M + 2 methionine when only M + 1 aspartate was present indicated that at least some glycine was produced via reverse Shmt to generate ^13^C-labeled C1 units, from the third carbon in serine, in MeTHF, which can be converted to 5-methyltetrahydrofolate (MTHF) and used to produce M + 2 methionine from M + 1 aspartate (Fig. 6A and Fig. S3A). ^13^C labeling of C1 units (9.2% M + 1 during [6-^13^C_1_]glucose labeling) in 10-formyltetrahydrofolate (FTHF) was also apparent from labeling patterns in purine nucleotides (Fig. S3C).

*T. saccharolyticum* has annotated genes encoding most of the enzymes required for isoleucine biosynthesis from threonine, but it does not have an annotated threonine deaminase, which is the first step in this pathway. Similarly, most of the enzymes required for isoleucine synthesis via citramalate are annotated, but citramalate synthase is missing (22, 23). Of these routes, the citramalate pathway (49) was necessary to explain isoleucine labeling patterns (Fig. 6B). During [1,2-^13^C_2_]glucose labeling, the production of M + 6 isoleucine can only be explained by citramalate pathway activity, and the close to equal proportions of M + 0 to M + 6 and M + 2 to M + 4 labeled forms also support isoleucine production via this pathway. Labeling using an equimolar mixture of [U-^13^C_6_]glucose and unlabeled glucose supported primary production of isoleucine via the citramalate pathway and indicated that less than 10% of isoleucine was synthesized from threonine (Fig. S3D).

### Reversibility of glycolytic reactions.

Having reconstructed the central metabolic network of *T. saccharolyticum*, we sought to compare the reversibility of glycolytic reactions in *T. saccharolyticum* to those in *C. thermocellum* to identify potential factors influencing the greater ethanol productivity in *T. saccharolyticum.* As previously detailed in the introduction, the glycolytic pathway in *C. thermocellum* has several features that set it apart from the canonical EMP glycolytic pathway, including distinct cofactor utilization at specific steps in the pathway and the use of alternative routes for the conversion of PEP to pyruvate (Fig. 1). We hypothesized that the nonstandard glycolytic pathway of *C. thermocellum* will display substantial differences in reaction reversibility and thermodynamics compared to those of the canonical EMP glycolytic pathway, such as that possessed by *T. saccharolyticum*. We used ^13^C and ^2^H steady-state labeling experiments to directly compare reaction reversibility between the glycolytic pathways of *C. thermocellum*, *T. saccharolyticum*, and anaerobically grown Escherichia coli as an additional point of reference.

### (i) Reversibility of upper glycolysis (glucose to glyceraldehyde-3-phosphate).

**FIG 7:**
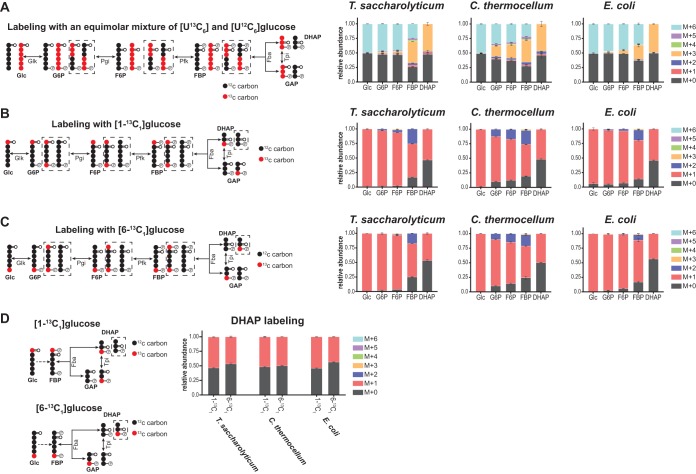
^13^C labeling reveals reversibility of upper glycolytic reactions. (A, B, and C) Steady-state labeling patterns of upper glycolytic intermediates in *C. thermocellum*, *T. saccharolyticum*, and E. coli during anaerobic growth on an equimolar mixture of [U-^13^C_6_]- and [U-^12^C_6_]glucose (A), [1-^13^C_1_]glucose (B), or [6-^13^C_1_]glucose (C). The production of M + 3 FBP in panel A or M + 2 and M + 0 FBP in panels B and C indicated high reversibility of the Fba reaction in all three bacteria. Large differences in the reverse propagation of M + 3 FBP (A) or M + 2 and M + 0 FBP (B and C) to F6P suggested that the Pfk reaction was substantially more reversible in *C. thermocellum* than *T. saccharolyticum* or E. coli. (D) The reversibility of triose phosphate isomerase (Tpi) was revealed by [6-^13^C_1_]glucose and [1-^13^C_1_]glucose labeling. During [6-^13^C_1_]glucose labeling, forward Fba activity first produces M + 0 DHAP. However, reverse Tpi flux results in the production of M + 1 DHAP from M + 1 GAP. Thus, the closer the M + 1 DHAP fraction is to 50%, the more reversible the Tpi. Labeling data indicated that Tpi was most reversible in *C. thermocellum*, with a proportion of M + 1 DHAP approaching 50% under both the [1-^13^C_1_]glucose and [6-^13^C_1_]glucose conditions. *T. saccharolyticum* had a less reversible Tpi reaction, followed by E. coli, which had a significant enrichment of M + 1 DHAP under the [1-^13^C_1_]glucose condition and an enrichment of M + 0 DHAP under the [6-^13^C_1_]glucose condition. The diagrams on the left depict predicted metabolite labeling. Solid arrows represent a single reaction; dashed arrows represent multiple reaction steps. ^13^C-labeled carbons are colored red; ^12^C-labeled carbons are colored black. Dashed boxes indicate metabolite-labeled forms produced by reverse flux. Data are averages from 2 or 3 independent biological replicates. Error bars show ±SD.

Labeling with an equimolar mix of [U-^13^C_6_]glucose (M + 6, all carbons are ^13^C labeled) and unlabeled glucose (M + 0, all carbons are ^12^C) revealed substantial differences in the reversibility of upper glycolytic reactions between *C. thermocellum*, *T. saccharolyticum*, and E. coli (Fig. 7). In this labeling experiment, forward phosphofructokinase activity (i.e., F6P→FBP) will first produce a 50:50 mixture of fully labeled (M + 6) and unlabeled (M + 0) FBP. However, as DHAP and GAP are ∼50% M + 0 and ∼50% M + 3 labeled, reverse flux from Fba will produce a 25:50:25 mixture of M + 6, M + 3, and M + 0 FBP. Therefore, the production of M + 3 FBP provides a measure of the *in vivo* reversibility of the Fba reaction. The fractions of M + 3 FBP in *C. thermocellum*, *T. saccharolyticum*, and E. coli were ∼38%, ∼37%, and ∼24%, respectively, indicating that Fba is highly reversible in all three organisms but significantly less so in E. coli (*P* < 0.05) (Fig. 7A). Once produced, M + 3 FBP may propagate back to F6P and G6P. Therefore, the reversibility of the upstream reactions with Pfk and phosphoglucose isomerase (Pgi; G6P→F6P) is informed by the production of M + 3 F6P and M + 3 G6P. The substantially larger M + 3 F6P fraction in *C. thermocellum* (∼23%) versus *T. saccharolyticum* (∼3%) and E. coli (∼5%) indicates that phosphofructokinase is substantially more reversible in this bacterium (*P* < 0.005). This observation is consistent with PP_i_-Pfk in *C. thermocellum* being less energetically favorable than the ATP-Pfk used by *T. saccharolyticum* and E. coli (20, 25, 50, 51). As shown in Fig. 7B and C, differences in the reversibility of the Pfk and Fba reactions across the three bacteria were also evident from the production of M + 0 and M + 2 FBP and F6P during [1-^13^C_1_]- and [6-^13^C_1_]glucose labeling.

Reversibility of triose phosphate isomerase (Tpi; DHAP→GAP) was revealed by [6-^13^C_1_]- and [1-^13^C_1_]glucose labeling (Fig. 7D). For example, during [6-^13^C_1_]glucose labeling, forward Fba activity first produces M + 0 DHAP and M + 1 GAP. Forward Tpi activity then produces M + 0 GAP from M + 0 DHAP. However, reverse Tpi flux results in the production of M + 1 DHAP from M + 1 GAP. Thus, the closer the M + 1 DHAP fraction is to 50%, the closer Tpi is to equilibrium (i.e., equal forward and reverse flux). The fractions of M + 1 DHAP in *C. thermocellum*, *T. saccharolyticum*, and E. coli were 49.8%, 46.4%, and 43.5%, respectively, indicating that Tpi is highly reversible in all three organisms. The data indicated that Tpi was significantly more reversible in *C. thermocellum* than E. coli (*P* < 0.005), although the difference between *C. thermocellum* and *T. saccharolyticum* did not reach statistical significance (*P* = 0.07).

### (ii) Reversibility of lower glycolysis (GAP to PEP).

^2^H tracers can provide information on the reversibility of reactions where a C-H bond is broken or formed via dehydration, isomerization, or dehydrogenation (27–29). Hence, we used [4-^2^H_1_]glucose and [5-^2^H_1_]glucose for investigating reversibility of lower glycolytic reactions (i.e., GAP to PEP) in which carbon rearrangements do not occur.

**FIG 8:**
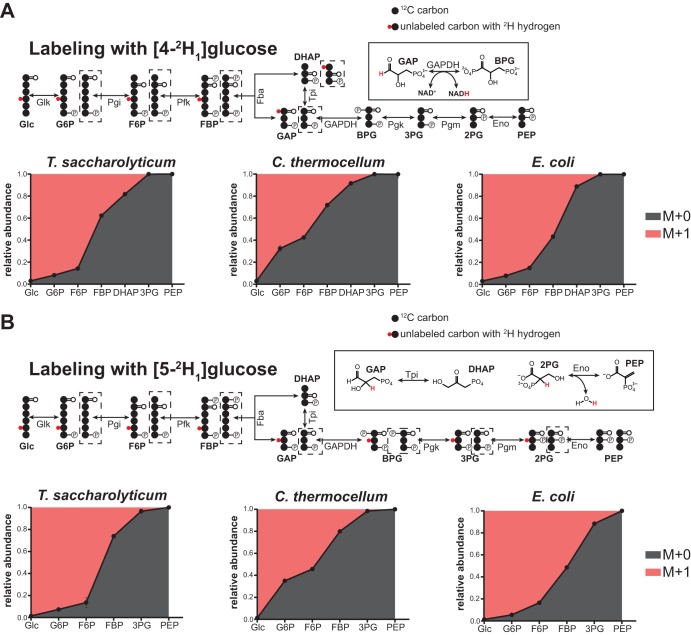
^2^H labeling reveals reversibility of upper and lower glycolytic reactions. Steady-state labeling patterns of lower glycolytic intermediates in *C. thermocellum*, *T. saccharolyticum*, and E. coli during anaerobic growth on [4-^2^H]glucose or [5-^2^H]glucose. (A) During [4-^2^H_1_]glucose labeling, GAPDH converts [1-^2^H_1_]GAP to unlabeled BPG (the deuteron is transferred to [^2^H_1_]NADH). Reverse GAPDH flux then can produce unlabeled GAP if unlabeled NADH is used by the enzyme. The M + 0 GAP produced thusly can propagate to upstream glycolytic intermediates and be used as a measure of the reversibility of GAPDH and upstream reactions. *C. thermocellum* displayed a substantially larger loss of ^2^H labeling (i.e., greater M + 0 fraction) across glycolytic intermediates (i.e., DHAP, FBP, F6P, and G6P) than *T. saccharolyticum* and E. coli, indicating increased reversibility of its glycolytic pathway. (B) During growth on [5-^2^H_1_]glucose, GAP, BPG, 3PG, and 2PG become deuterated at the second carbon. Conversion of [2-^2^H_1_]2PG to unlabeled PEP results in the loss of deuterium to water. The propagation of unlabeled PEP to upstream intermediates then indicates the reversibility of upstream reactions. The deuterium in [2-^2^H_1_]GAP also may be lost during its conversion to DHAP. *C. thermocellum* displayed a substantially larger loss of ^2^H labeling across all glycolytic intermediates than *T. saccharolyticum* and E. coli, indicating once again the increased reversibility of its glycolytic reactions. The diagrams on the left depict predicted metabolite labeling. Solid arrows represent a single reaction; dashed arrows represent multiple reaction steps. ^12^C carbons are colored black; deuterons (^2^H) are depicted as small red circles. Dashed boxes indicate metabolite-labeled forms produced via reverse flux. The chemical reactions enclosed in solid boxes in panels A and B illustrate the step in the pathway at which the loss of deuterium occurs. Data are averages from 2 or 3 independent biological replicates. Error bars show ±SD. Error bars smaller than the icon used to represent the average are not visible.

As shown in Fig. 8A, during [4-^2^H_1_]glucose labeling, [1-^2^H_1_]GAP (i.e., GAP with a deuterium atom bound to its first carbon) is produced via Fba. Conversion of [1-^2^H_1_]GAP to 1,3-bisphosphoglycerate (BPG), catalyzed by glyceraldehyde-3-phosphate dehydrogenase (GAPDH), removes the deuterium from [1-^2^H_1_]GAP and places it on NAD^+^, generating ^2^H-labeled NADH. Reverse GAPDH flux then can produce unlabeled GAP if unlabeled NADH is used. The M + 0 GAP produced can propagate to DHAP (via reverse Tpi) and FBP (via reverse Fba), as well as to other upstream glycolytic intermediates. We observed that during growth on [4-^2^H_1_]glucose, *C. thermocellum* displayed a larger loss of ^2^H labeling across all glycolytic intermediates than *T. saccharolyticum* and E. coli, which indicated increased reversibility of glycolytic reactions in this bacterium. For example, although we could not reliably measure GAP, higher fractions of M + 0 DHAP and M + 0 FBP in *C. thermocellum*, i.e., 92% and 72%, respectively, versus 82% and 62% in *T. saccharolyticum* or 89% and 43% M + 0 in E. coli, suggested greater reversibility of its GAPDH/Tpi and GAPDH/Fba pairs of reactions (*P* < 0.05 for all pairwise comparisons).

During growth on [5-^2^H_1_]glucose, the intermediates GAP, BPG, 3PG, and 2-phosphoglycerate (2PG) become deuterated at the second carbon (Fig. 8B). The loss of deuterium can occur from the conversion of [2-^2^H_1_]GAP to M + 0 DHAP via reverse Tpi or by the forward phosphopyruvate hydratase reaction (Eno) converting [2-^2^H_1_]2PG to M + 0 PEP. Once produced, propagation of M + 0 DHAP or M + 0 PEP to upstream metabolites provides a measure of the reversibility of Tpi, Eno, and other glycolytic reactions. As with [4-^2^H_1_]glucose, the greater loss of ^2^H labeling across glycolytic intermediates in *C. thermocellum* during [5-^2^H_1_]glucose labeling indicated increased reversibility of glycolysis in this bacterium compared to that of *T. saccharolyticum* or E. coli. For example, taking Tpi reversibility into account (Fig. 7D), the larger fractions of M + 0 3PG in *C. thermocellum* (98.4%) and *T. saccharolyticum* (96.6%) suggested increased reversibility of the Pgm/Eno reaction pair in these thermophilic organisms compared to that in E. coli (88.4%; *P* < 0.05 for both pairwise comparisons).

### (iii) Reversibility of the conversion of phosphoenolpyruvate to pyruvate.

*C. thermocellum* lacks pyruvate kinase (Pyk; PEP + ADP→Pyr + ATP) and instead transforms PEP to Pyr via pyruvate phosphate dikinase (Ppdk; PEP + AMP + PP_i_→Pyr + ATP + P_i_) or the malate shunt (Fig. 5). In contrast, Pyr production from PEP in E. coli and *T. saccharolyticum* occurs via Pyk or the phosphotransferase system (PTS) (18, 40, 52, 53). On its way toward fermentative pathways, Pyr is decarboxylated by Pfor or Pfl in *C. thermocellum* and *T. saccharolyticum* (6, 24, 54–57). In anaerobically grown E. coli, Pyr is converted to acetyl-CoA by Pfl (58, 59).

When cells are grown in an equimolar mixture of [U-^13^C_6_]glucose and unlabeled glucose, the Pyr initially produced via glycolysis is either M + 0 or M + 3, and its decarboxylation produces acetyl-CoA containing an M + 0 or M + 2 acetyl group. Complete or partial (i.e., decarboxylation step only) reversibility of Pfor or Pfl resulted in the interchange of the carboxylic group in Pyr with unlabeled CO_2_ or formate to produce M + 2 Pyr (60) (Fig. 5). Pyr decarboxylation was significantly more reversible in E. coli (33.2% M + 2 Pyr) and *C. thermocellum* (26.5% M + 2 Pyr) than in *T. saccharolyticum* (14.8% M + 2 Pyr; *P* < 0.05 for both pairwise comparisons).

Once formed, M + 2 Pyr may propagate to upstream metabolites to produce M + 2 PEP, indicating reversibility of PEP-to-Pyr conversion by whichever route the organism has available. Despite a potentially longer path, we observed that PEP-to-Pyr conversion was significantly more reversible (*P* < 0*.01*) in *C. thermocellum* (10.2% M + 2 PEP) than in E. coli (2.0% M + 2 PEP). Data suggested that PEP-to-Pyr conversion was also more reversible in *C. thermocellum* than *T. saccharolyticum* (3.9% M + 2 PEP), but this difference was not significant at *P* < 0.05. Considering the reported absence of pyruvate carboxylase or oxaloacetate decarboxylase activity in *C. thermocellum* (18, 26), the production of M + 2 Mal (14.0%) and M + 2 aspartate (12.0%; surrogate for OAA) suggested considerable reversibility of the malate shunt. Another potential alternative for the production of M + 2 aspartate and M + 2 Mal in *C. thermocellum* is a reversible citrate synthase, which has been shown to be reversible in other thermophilic bacteria, such as Desulfurella acetivorans and Thermosulfidibacter takaii (61, 62).

### (iv) Reversibility of ethanol production from pyruvate.

**FIG 9:**
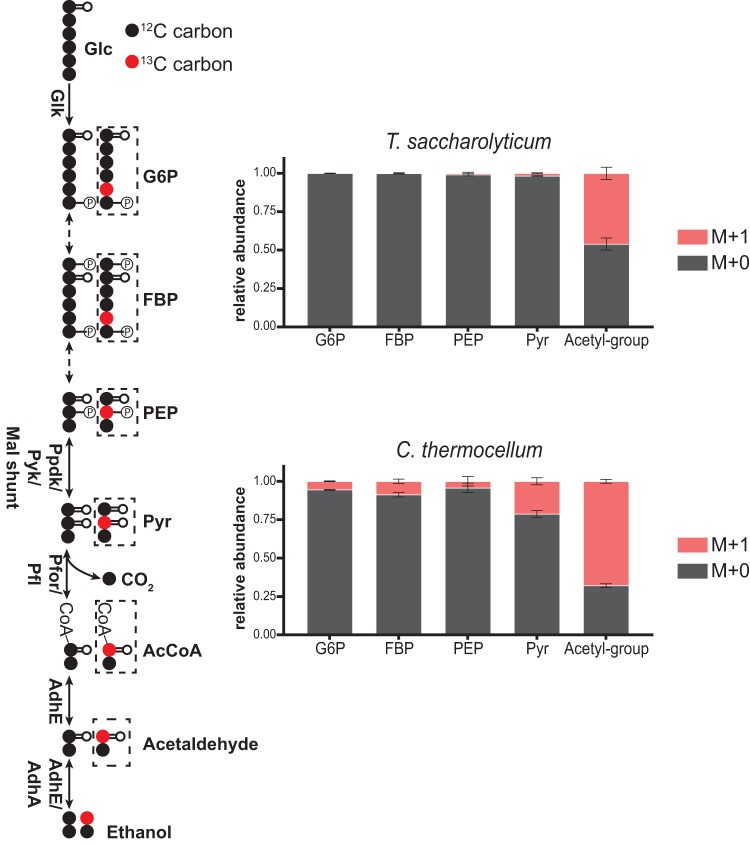
Reversibility of the pyruvate-to-ethanol pathway in *C. thermocellum* and *T. saccharolyticum.* Steady-state labeling with [1-^13^C_1_]ethanol in *C. thermocellum* and *T. saccharolyticum* revealed that the pyruvate-to-ethanol pathway in *C. thermocellum* was substantially more reversible than that of *T. saccharolyticum.* Reversibility of the AdhA and AdhE reactions resulted in ^13^C-labeled AcCoA (at the acetyl group), which was more labeled in *C. thermocellum* than in *T. saccharolyticum.* Reverse activity of Pfor or Pfl resulted in the production of M + 1 pyruvate. Reverse activity of EMP reactions above pyruvate resulted in the production of labeled glycolytic intermediates. The diagrams on the left depict predicted metabolite labeling. Solid arrows represent a single reaction; dashed arrows represent multiple reaction steps. ^13^C-labeled carbons are colored red; ^12^C-labeled carbons are colored black. Dashed boxes indicate metabolite-labeled forms produced by reverse flux. Data are averages from 3 independent biological replicates. Error bars show ±SD. Abbreviations: AdhA, alcohol dehydrogenase; AdhE, bifunctional alcohol/aldehyde dehydrogenase.

Since *C. thermocellum* and *T. saccharolyticum* are being developed for the production of ethanol from lignocellulosic biomass, we were interested in investigating the reversibility of their ethanol fermentation pathways in addition to glycolysis. In both *C. thermocellum* and *T. saccharolyticum*, acetyl-CoA, produced from Pyr, is reduced to acetaldehyde [AcCoA + NAD(P)H→acetaldehyde + NAD(P)^+^] and then to ethanol [acetaldehyde + NAD(P)H→ethanol + NAD(P)^+^] by a bifunctional aldehyde/alcohol dehydrogenase, AdhE (15). *T. saccharolyticum* also possesses an NADPH-dependent alcohol dehydrogenase (AdhA) (16). To investigate the reversibility of the Pyr-to-ethanol pathway, we grew *C. thermocellum* and *T. saccharolyticum* in the presence of [1-^13^C_1_]ethanol and unlabeled cellobiose (for *C. thermocellum*) or unlabeled glucose (for *T. saccharolyticum*). The incorporation of ^13^C from [1-^13^C_1_]ethanol into acetyl-CoA was significantly larger (*P* < 0.005) in *C. thermocellum* (67.9% M + 1) than *T. saccharolyticum* (46.0% M + 1), indicating increased reversibility of the conversion of acetyl-CoA to ethanol in *C. thermocellum* (Fig. 9). Additionally, Pyr was 21.3% M + 1 labeled in *C. thermocellum* but only 1.7% M + 1 labeled in *T. saccharolyticum*, indicating a significantly less reversible Pfor/Pfl reaction in *T. saccharolyticum* (*P* < 0.01). We also observed significant propagation of ^13^C from [1-^13^C_1_]ethanol into upper glycolytic intermediates, such as G6P (5.5% M + 1) and FBP (8.8% M + 1), in *C. thermocellum* (*P* < 0.05 for both metabolites). However, these metabolites remained essentially unlabeled in *T. saccharolyticum* (<0.2% M + 1). Overall, this analysis indicated a substantially more reversible ethanol fermentation pathway in *C. thermocellum* than *T. saccharolyticum.*

### ^2^H and ^13^C metabolic flux analysis.

**FIG 10:**
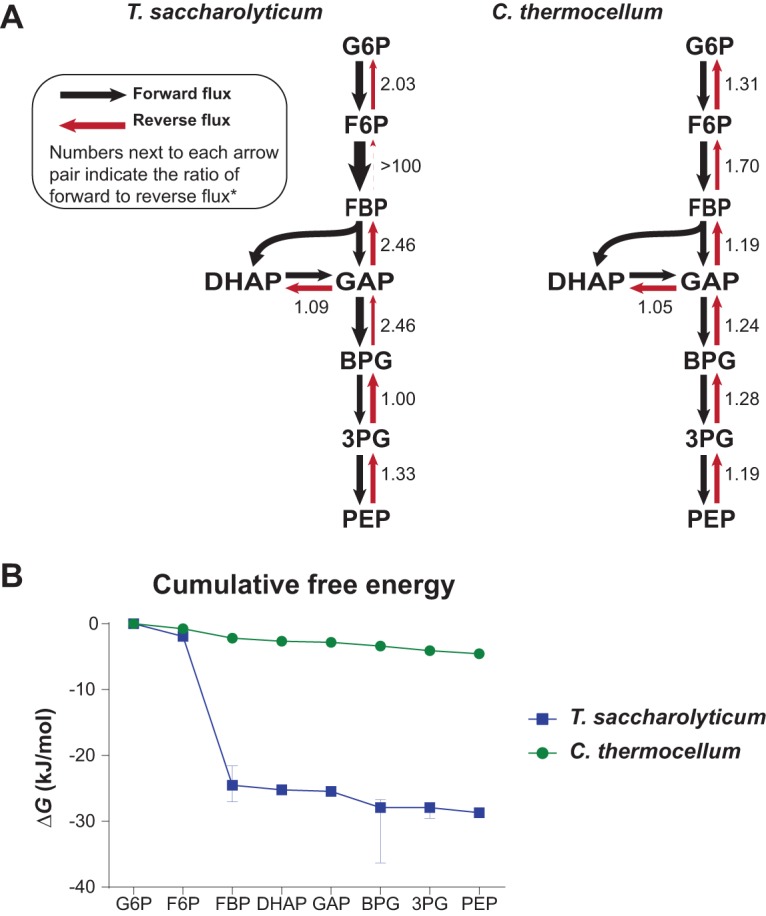
Glycolytic reversibility in *C. thermocellum* and *T. saccharolyticum.* (A) Flux maps for *C. thermocellum* and *T. saccharolyticum* during glucose fermentation were generated by simultaneously fitting data from five parallel ^13^C and ^2^H tracer experiments (i.e., [1-^13^C_1_]glucose, [6-^13^C_1_]glucose, [4-^2^H_1_]glucose, [5-^2^H_1_]glucose, and an equimolar mixture of [U^13^C_6_]glucose and unlabeled glucose) into a single, statistically acceptable flux map. Arrow thickness is proportionate to net flux. Numbers next to each arrow represent net flux, with reverse fluxes in parentheses. Fluxes are represented as a proportion of glucose uptake, which was normalized to 100. Malic enzyme was omitted from the *C. thermocellum* model due to the analysis being unable to resolve flux through malic enzyme versus Ppdk. Omission of malic enzyme had a negligible effect on flux ratios in glycolysis (see Materials and Methods). *, see Table S2 for complete MFA results, including 95% confidence intervals. (B) MFA-derived Δ*G* values for glycolytic reactions (Table 1) revealed that the glycolytic pathway in *C. thermocellum* was remarkably close to thermodynamic equilibrium, with an overall drop in Gibbs free energy of −4.55 kJ/mol between G6P and PEP. In contrast, the overall drop in free energy of glycolysis in *T. saccharolyticum* was substantially greater, Δ*G* = −28.67 kJ/mol. The limited thermodynamic driving force of the glycolytic pathway in *C. thermocellum* could be attributed in large part to the small driving force of the Pfk reaction, which had a Δ*G* of only −1.45 kJ/mol compared to −22.57 kJ/mol in *T. saccharolyticum*. Intracellular metabolite concentrations provided cross-validation of Δ*G* estimates for the Pfk reaction in *T. saccharolyticum* (see Materials and Methods). Error bars shown represent the 95% confidence intervals of the free energy estimates. Error bars smaller than the icon used to represent the average are not visible.

To determine *in vivo* flux ratios (*J^+^/J^–^*) and compare the thermodynamics of the distinct glycolytic pathways of *C. thermocellum* and *T. saccharolyticum*, we integrated data from five ^13^C and ^2^H tracer experiments ([1-^13^C_1_]-, [6-^13^C_1_]-, [4-^2^H_1_]-, [5-^2^H_1_]glucose, and an equimolar mixture of [U-^13^C_6_]glucose and unlabeled glucose) into a single flux map for each organism (Fig. 10A and Table S2). MFA was performed using the INCA software suite, which utilizes the elementary metabolite unit (EMU) framework to model isotopic distributions (63, 64). Reaction directionalities were not predetermined for most reactions in central carbon metabolism (glycolysis, TCA cycle, and PPP) to allow combined ^2^H and ^13^C MFA to determine forward and reverse fluxes. Reaction directionality was preassigned primarily for transport reactions, dilution fluxes, and combined amino acid biosynthesis reactions.

10.1128/mSystems.00736-19.6 Table S2 Integrated ^13^C and ^2^H MFA-derived fluxes for *T. saccharolyticum* and *C. thermocellum*. Download Table S2, XLSX file, 0.03 MB. Copyright © 2020 Jacobson et al. 2020 Jacobson et al. This content is distributed under the terms of the Creative Commons Attribution 4.0 International license.

For *T. saccharolyticum*, ^2^H and ^13^C MFA best-fit solutions validated the qualitative assessment of its metabolic network, presented earlier. We constructed alternative models containing all possible glycolytic routes (e.g., EMP, ED, and oxidative pentose phosphate pathways) as well as models lacking reactions that we identified as necessary to explain labeling patterns of specific metabolites (e.g., citramalate pathway for isoleucine production and SBP bypass for S7P production). The results of this analysis agreed with our previous conclusions and predicted an inactive ED pathway (i.e., <0.25% glycolytic flux), an inactive oxidative PPP (i.e., 0% flux), and a bifurcated TCA cycle with oxidative production of AKG and reductive production of succinate. Although Pepck typically is considered a gluconeogenic reaction (i.e., transforming OAA to PEP), our MFA results supported the activity of a PEP carboxylating enzyme producing OAA from PEP. We also found that the SBP bypass was necessary to explain labeling patterns in PPP intermediates and aromatic amino acids. This analysis also supported qualitative conclusions regarding amino acid biosynthetic routes, such as isoleucine synthesis via the citramalate pathway instead of from threonine. Additionally, the production of serine from 3PG and its consumption by Shmt to form labeled THF intermediates was necessary to achieve a statistically acceptable model fit.

For *C. thermocellum*, our MFA results were also consistent with the previously reported central metabolic network (26). Specifically, our analysis predicted glycolysis via the EMP pathway with no ED pathway activity, a solely nonoxidative PPP containing a SBP bypass, and a bifurcated TCA cycle. Our results were also consistent with reported amino acid synthesis pathways in *C. thermocellum*.

### Comparative thermodynamic pathway analysis.

Using the relation *ΔG = −RT* ln(*J^+^/J^−^*), we estimated *ΔG* of glycolytic reactions (i.e., from G6P to PEP) from MFA-derived flux ratios (*J^+^/J*^−^) in *C. thermocellum* and *T. saccharolyticum* (Fig. 10B and Table 1). For a small number of reactions, we used intracellular metabolite concentrations to improve *ΔG* estimates obtained from MFA-derived flux ratios (Table 1). Estimated *ΔG* values aligned with the qualitative assessment of reaction reversibility presented above and showed that the glycolytic pathway in *C. thermocellum*, with an overall *ΔG* of −4.55 kJ/mol, operates remarkably close to thermodynamic equilibrium. In comparison, the glycolytic pathway in *T. saccharolyticum* was substantially more thermodynamically favorable (overall *ΔG* = −28.67 kJ/mol) and was comparable to the glycolytic pathway in anaerobically grown E. coli (overall *ΔG* = −23.47 kJ/mol) (29) (Fig. S4).

**TABLE 1:** Reaction free energies calculated from integrated ^13^C and ^2^H MFA-derived fluxes in *C. thermocellum* and *T. saccharolyticum*

Glycolytic reaction	*ΔG* in:					
	*C. thermocellum*			*T. saccharolyticum*		
	Best fit	LB^*b*^	UB^*b*^	Best fit	LB	UB
G6P + H↔F6P + H	−0.74	−0.67	−0.81	−1.93	−1.62	−2.25
F6P↔FBP^*a*^	−1.45	−1.40	−1.49	−22.57	−19.67	−25.11
FBP + H↔DHAP + GAP	−0.47	−0.45	−0.50	−0.70	−0.43	−1.18
DHAP + H↔GAP + H	−0.14	−0.12	−0.16	−0.24	−0.20	−0.30
GAP + NAD↔BPG + NADH	−0.59	−0.20	−1.14	−2.46	−1.25	−10.87
BPG↔3PG	−0.67	−0.13	−1.07	0.00	0.00	−1.63
3PG↔PEP + H	−0.48	−0.30	−0.71	−0.77	−0.51	−1.26

a The F6P↔FBP reaction free energy for *T. saccharolyticum* is calculated from our measured intracellular metabolite concentrations, because the MFA-derived free energy was unbounded in the negative direction.

b LB and UB are the lower and upper bounds defined by a 95% confidence interval for the free energy.

10.1128/mSystems.00736-19.4 FIG S4 Thermodynamic profile of glycolysis in *C. thermocellum*, *T. saccharolyticum*, E. coli, and Z. mobilis. We compared the free energy of glycolysis in *C. thermocellum* and *T. saccharolyticum* to published thermodynamic profiles for anaerobically grown E. coli (19) and Z. mobilis (17)*. T. saccharolyticum* and anaerobically grown E. coli have glycolytic pathways with similar thermodynamic profiles. The glycolytic pathway of Z. mobilis is, in contrast, substantially more thermodynamically favorable. The free energy of the Pgi and Pfk reactions in E. coli were previously reported as a single lumped reaction (Pgi-Pfk), so we calculated the free energy of the Pfk reaction using our measured intracellular metabolite concentrations. We used our calculated *ΔG* range for the Pfk reaction and assigned the remainder of the *ΔG* of the Pgi-Pfk combined reaction to Pgi. Error bars shown represent the 95% confidence intervals of the free energy estimates. Error bars smaller than the icon used to represent the average are not visible. Download FIG S4, PDF file, 0.4 MB. Copyright © 2020 Jacobson et al. 2020 Jacobson et al. This content is distributed under the terms of the Creative Commons Attribution 4.0 International license.

The limited thermodynamic driving force of the glycolytic pathway in *C. thermocellum* could be attributed in large part to the small driving force of the Pfk reaction, which had a *ΔG* of only −1.45 kJ/mol compared to −22.57 kJ/mol in *T. saccharolyticum* and −18.84 kJ/mol in E. coli. The small *ΔG* of Pfk in *C. thermocellum* was likely due to the use of PP_i_ instead of ATP as the high-energy phosphate donor. Indeed, the standard free energy for the ATP-dependent Pfk (*ΔG* = −15.0 kJ/mol) is ∼10.6 kJ/mol more favorable than the PP_i_-dependent Pfk (*ΔG* = −4.4 kJ/mol) due to the greater free energy of ATP hydrolysis than PP_i_ hydrolysis (50).

Consistent with our qualitative observations regarding its reversibility, the Fba reaction operated close to thermodynamic equilibrium in both *T. saccharolyticum* (−0.70 kJ/mol) and *C. thermocellum* (−0.47 kJ/mol) but was more thermodynamically favorable in anaerobic E. coli (−3.64 kJ/mol) (29). Also in agreement with qualitative observations, the Pgi reaction was slightly more favorable in *T. saccharolyticum* (−1.93 kJ/mol) than in *C. thermocellum* (−0.74 kJ/mol). The *ΔG* of lower glycolytic reactions (GAPDH to Eno) were close to equilibrium in both *C. thermocellum* and *T. saccharolyticum* but were generally more favorable in *T. saccharolyticum*. For example, the *ΔG* of the Pgm/Eno pair of reactions was −0.49 kJ/mol in *C. thermocellum* compared to −0.77 kJ/mol in *T. saccharolyticum*.

In both *C. thermocellum* and *T. saccharolyticum*, most of the reactions in the PPP, including Sba, SBPase, Rpi, and Rpe, as well as the transketolase half reactions TktA (Xu5P→GAP + C2) and TktB (S7P→R5P + C2), all had *ΔG* no more favorable than −1.3 kJ/mol, putting them close to thermodynamic equilibrium (Table S3).

10.1128/mSystems.00736-19.7 Table S3 Reaction free energies calculated from simultaneous ^13^C and ^2^H MFA-derived fluxes in *C. thermocellum* and *T. saccharolyticum*. Download Table S3, XLSX file, 0.01 MB. Copyright © 2020 Jacobson et al. 2020 Jacobson et al. This content is distributed under the terms of the Creative Commons Attribution 4.0 International license.

## DISCUSSION

### Metabolite concentrations and thermodynamics of glycolysis.

Intracellular concentrations of glycolytic intermediates across the bacteria in this study reflected the thermodynamic profile of their glycolytic pathways (see Table S1). For example, the limited driving force of the PP_i_-Pfk reaction in *C. thermocellum* was associated with a low FBP concentration, which was about one-third of that in *T. saccharolyticum* or E. coli. In contrast, G6P and F6P levels in *C. thermocellum* were higher than those in *T. saccharolyticum* (6.8-fold and 1.2-fold, respectively) and also higher than those in E. coli (4.1-fold and 5.0-fold, respectively). Higher levels of G6P and F6P in *C. thermocellum* potentially reflect the need to accumulate larger amounts of upper glycolytic intermediates to drive the PP_i_-Pfk reaction forward. Indeed, the FBP/F6P ratio was considerably lower in *C. thermocellum* (∼1) than E. coli (∼15) or *T. saccharolyticum* (∼3.7). Thus, higher intracellular FBP concentrations maintained by the more favorable upper EMP reactions in E. coli and *T. saccharolyticum* may help drive subsequent reactions in glycolysis.

Interestingly, ATP/ADP and GTP/GDP ratios were considerably lower in *C. thermocellum* (25.7 and 28.7, respectively) than E. coli (66.0 and 42.4) and *T. saccharolyticum* (59.9 and 61.4). The higher ATP/ADP ratios in E. coli and *T. saccharolyticum* likely contribute to the favorability of the ATP-Pfk reaction and other reactions in upper glycolysis in these organisms. In contrast, the low ATP/ADP and GTP/GDP ratios in *C. thermocellum* may help make ATP/GTP-generating reactions in lower glycolysis, such as GAPDH and Ppdk, more favorable but may also slow biosynthetic reactions that rely on ATP/GTP hydrolysis, such as amino acid, protein, fatty acid, nucleotide, and mRNA synthesis.

### Enzyme efficiency, metabolic flux, and product titers.

Thermodynamics constitutes a key determinant of enzyme efficiency and flux within metabolic pathways. As determined by *ΔG = −RT* ln(*J^+^/J^−^*), enzyme efficiency (i.e., the fraction of enzyme used in the forward versus reverse direction) is directly proportional to the drop in Gibbs free energy (*ΔG*) of a biochemical reaction (31, 32). Thus, the fraction of enzyme that counterproductively catalyzes the reverse reaction (*J^−^*) increases exponentially as *ΔG* approaches equilibrium, decreasing the net reaction rate (*J^+^*– *J^−^*) (33, 65). This implies that a metabolic pathway that is close to thermodynamic equilibrium will require substantially greater amounts of catalytically active enzyme to maintain a given flux than a more thermodynamically favorable pathway. Therefore, considering cellular limits on total enzyme abundance (66), the small thermodynamic driving force of *C. thermocellum* glycolysis could represent a limiting factor for engineering ethanologen strains with high glycolytic rates.

Besides limiting flux, a small thermodynamic driving force also can make a pathway more susceptible to end product inhibition, since only a comparatively small amount of product would need to accumulate before some reaction within the pathway approaches thermodynamic equilibrium (*ΔG*→0) and effectively stops further substrate utilization and product formation. Therefore, it is likely that the limited thermodynamic driving force of the *C. thermocellum* glycolytic pathway represents a key contributor to its low ethanol titer (25 g/liter) (6) compared to that of organisms with more thermodynamically favorable glycolysis, such as *T. saccharolyticum* (70 g/liter) (3), S. cerevisiae (75 g/liter) (67), and Z. mobilis (>85 g/liter) (27, 68, 69). Indeed, a recent computational evaluation that used maximum-minimum driving force (MDF)-based analysis to model the thermodynamics of the glycolytic and ethanol fermentation pathways in *C. thermocellum* predicted that the thermodynamic driving force of these pathways is rapidly depleted as ethanol accumulates extracellularly (70). This limitation may help explain the difficulties encountered by groups attempting to engineer *C. thermocellum* to increase product titers compared to the successes at engineering *T. saccharolyticum* (6, 10, 13, 16, 70).

Our results suggest that a promising strategy for increasing glycolytic flux and ethanol titers in *C. thermocellum* is to replace PP_i_-Pfk with an ATP-dependent Pfk. In addition, replacing the malate shunt and Ppdk routes for conversion of PEP to Pyr with Pyk also may increase flux and ethanol titers by providing a single more thermodynamically favorable route. Indeed, *C. thermocellum* strains expressing an exogenous Pyk achieve higher ethanol titers (13), and elimination of malate shunt activity improved ethanol production in Clostridium cellulolyticum, another cellulolytic ethanol producer (71). Finally, adding a mechanism for ATP hydrolysis could increase the thermodynamic favorability of ATP-harvesting reactions in glycolysis (Pgk and Pyk) (31). ATP demand has been implicated in glycolytic flux control in a number of organisms, including Z. mobilis, S. cerevisiae, and E. coli. (72–74). This strategy has a potential additional benefit in that it may limit cellular resources that are spent on replication by limiting access of biosynthetic reactions to ATP (72, 74).

The previously reported MDF-based analysis of *C. thermocellum* also produced a computationally derived thermodynamic profile of glycolysis (60). Our *in vivo* measurement of pathway free energies agreed with the general thermodynamic profile produced by MDF, with most of the free energy released by the Pfk and Ppdk reactions and the rest of the reactions closer to equilibrium. Although the overall profiles were similar, their analysis predicted more favorable PP_i_-Pfk (−6.67 kJ/mol) and Ppdk (−6.92 kJ/mol) reactions than the values we report in this study (−1.45 and −3.35 kJ/mol, respectively). This discrepancy may be due to *in vivo* constraints not accounted for in the MDF analysis, such as limits on enzyme abundance and the need to maintain adequate metabolite pools to drive biosynthetic pathways (31, 33, 34). In addition, the cellular objective in *C. thermocellum* may not be completely aligned with the objective function implicit in the MDF algorithm (i.e., to maximize the lowest free energy given a set of reaction steps).

### A trade-off between thermodynamic driving force and energy yield.

Interestingly, the limited driving force of the glycolytic pathway in *C. thermocellum* mirrors that of *C. cellulolyticum*, another cellulose-degrading organism that also utilizes PP_i_-Pfk, Ppdk, and the malate shunt (29, 71, 75). A potential advantage of glycolytic pathways with low thermodynamic driving force is increased energy efficiency, i.e., increased ATP or GTP yield per glucose. The use of alternative enzymes, such as PP_i_-Pfk (which uses PP_i_ in place of ATP) and Ppdk (which produces ATP from AMP and PP_i_), by cellulolytic microbes represents a potential mechanism for increased energy yield. However, recent research suggests that the PP_i_ needed for glycolysis is in excess of what can be generated as a byproduct of biosynthetic pathways and is instead generated by ATP-consuming glycogen cycling reactions (20). This casts some doubt on the use of PP_i_-dependent enzymes as a mechanism of ATP conservation. However, PP_i_ also could be generated by a membrane-bound proton-pumping pyrophosphatase (PP_i_ase, *Cthe_1425*), which is transcribed and expressed in *C. thermocellum* (24, 37). The proton gradient required for this process may originate from ferredoxin oxidation by an ion-tranlocating reduced ferredoxin:NAD^+^ oxidoreductase (Rnf) (76, 77), with reduced ferredoxin provided by Pfor in the ethanol fermentation pathway (12, 18). Thus, Pfor, Rnf, and PP_i_ase may work together to harvest additional energy from glycolysis by coupling pyruvate decarboxylation to proton translocation and subsequent PP_i_ production. Therefore, PP_i_ generation from a proton gradient represents a potential mechanism for energy conservation and increased ATP yield by a pathway that utilizes PP_i_-dependent enzymes.

The use of near-equilibrium glycolytic pathways with increased ATP yield by cellulolytic bacteria may represent an evolutionary adaptation to growth on cellulosic substrates. Specifically, microbes utilizing soluble substrates can maximize either the specific substrate consumption rate (grams of substrate × gram cells^−1^ hour^−1^) or the cell yield (grams of cells × gram substrate^−1^), the product of which is the specific growth rate (per hour). For microbes growing on cellulosic biomass, however, the specific substrate consumption rate is highly constrained, leading to strong selective pressure for cellulolytic bacteria to maximize the cell yield by increasing glycolytic ATP yield. Conversely, the use of highly thermodynamically favorable pathways with lower ATP yield, such as the ED pathway in Z. mobilis, likely represents an adaptation to glucose-rich environments (27, 31, 78).

In conclusion, our analysis revealed that the glycolytic pathway in *C. thermocellum* is remarkably close to thermodynamic equilibrium compared to the glycolytic pathways of *T. saccharolyticum* and E. coli. The primary contributor to the large reversibility of glycolysis in *C. thermocellum* is the Pfk reaction, but AdhE in the ethanol fermentation pathway is likely also an important contributor. Our findings help explain the low ethanol titer in *C. thermocellum* and suggest engineering strategies that can be used to increase its ethanol productivity and glycolytic rate.

## MATERIALS AND METHODS

### Materials.

Tracers ([1-^13^C_1_]glucose [98 to 99 atom% ^13^C], [6-^13^C_1_]glucose [99 atom% ^13^C], [1,2-^13^C_2_]glucose [99 atom% ^13^C], [U-^13^C_6_]glucose [99 atom% ^13^C], [4-^2^H_1_]glucose [98 atom% ^2^H], [5-^2^H_1_]glucose [98 atom% ^2^H], and [1-^13^C_1_]ethanol [99 atom% ^13^C]) were purchased from Cambridge Isotope Laboratories (Andover, MD). All other chemicals were purchased from Sigma-Aldrich (St. Louis, MO).

### Strains and growth conditions.

Wild-type *C. thermocellum* (DSM1313) was obtained from the DSMZ culture collection. Wild-type *T. saccharolyticum* (JW/YS-485L) was obtained courtesy of Juergen Wiegel, Department of Microbiology, University of Georgia. Fermentations and growth were carried out in MTC (11.56 g/liter morpholine propanesulfonic acid [MOPS] sodium salt, 3 g/liter trisodium citrate, 1.5 g/liter KH_2_PO_4_, 1.5 g/liter NH_4_SO_4_, 2.6 g/liter MgCl_2_·6H_2_O, 0.13 g/liter CaCl_2_·2H_2_O, 0.001 g/liter FeCl_2_·4H_2_O, 0.5 g/liter l-cysteine HCl·H_2_O, 0.004 g/liter *p*-aminobenzoic acid, 0.002 g/liter biotin, 0.002 g/liter cobalamin, 0.004 g/liter thiamine, and 5 g/liter either glucose or cellobiose; pH 7 for *C. thermocellum* and pH 6.8 for *T. saccharolyticum*).

We grew small cultures (7 to 10 ml) in 15-ml modified Hungate tubes for labeling experiments, intracellular metabolite quantitation, and growth curves. Larger cultures for extracellular metabolite quantitation were grown in 125- to 200-ml pressure bottles, with culture volume never exceeding more than 2/3 the volume of the bottle. Tubes or bottles were filled with medium base containing MOPS solution, sealed with butyl rubber stoppers, made anaerobic using a vacuum manifold, overlaid with N_2_ gas (oxygen scrubbed), and autoclaved. Other medium components were prepared, made anaerobic, autoclaved separately, and then added to culture tubes. Before manipulating cultures, syringes were made anoxic by repeatedly drawing and expelling headspace from an anaerobic sealed bottle containing 2.5% cysteine HCl solution. Tubes were placed in a 55°C water bath after inoculation for growth. Growth was quantified by the optical density at 600 nm (OD_600_) using a Genesys 20 UV-visible spectrophotometer equipped with a tube adapter. Inoculation and sampling of cultures took place using anoxic syringes, and any manipulation that required opening the tube or extended handling took place in an anaerobic chamber (Coy Laboratory) with a 5% CO_2_, 5% H_2_, 90% N_2_ atmosphere under anaerobic conditions (<100 ppm O_2_).

Escherichia coli RL3000 (MG1655 *IlvG^+^ rph^+^ pyrE^+^ ΔglcB*) was obtained courtesy of Robert Landick, Department of Bacteriology, University of Wisconsin–Madison (79). All growth, labeling, and metabolite quantitation were carried out in M9 minimal medium (6 g/liter Na_2_HPO_4_, 3 g/liter NaH_2_PO_4_, 0.5 g/liter NaCl, 1 g/liter NH_4_Cl, 4 g/liter glucose, 0.1 mM CaCl_2_, 2 mM MgSO_4_, 0.075 mM FeCl_3_) at 37°C in stirred flasks in an anaerobic chamber.

### Metabolite collection.

Working cultures were inoculated anaerobically with a 1:20 or greater dilution from overnight anaerobic cultures containing the same carbon source to an initial OD_600_ of ∼0.05. Cells were grown to mid-exponential phase (OD_600_ of 0.40 to 0.5), and then intracellular metabolites were collected inside the anaerobic chamber by vacuum filtration of 5 ml of culture through 0.45-μm hydrophilic nylon filters to separate cells from the media. Filters then were placed cell-side down in 1.5 ml of extraction solvent (40% acetonitrile, 40% methanol, and 20% water) and kept on dry ice to quench metabolism and extract metabolites. Cells were washed off the filter using the solvent, which was collected, vortexed, and then centrifuged for 5 min at 4°C to remove cellular debris, and the supernatant was collected for liquid chromatography-mass spectrometry (LC-MS) analysis. Labeling with positionally labeled ^13^C and ^2^H tracers was performed in biological duplicate.

### LC-MS of intracellular metabolites.

LC-MS analysis was carried out as described previously (27, 80). Chromatographic separation occurred on a 2.1- by 100-mm Acquity ultrahigh-pressure liquid chromatography ethylene bridged hybrid (UHPLC BEH) C_18_ column with 1.7-μm particle size (Waters) at 25°C on a Vanquish UPLC coupled to a Q Exactive Orbitrap high-resolution mass spectrometer (ThermoScientific) using an electrospray ionization source operating in negative mode. Intracellular metabolites were dried out of extraction solvent using N_2_ and resuspended in solvent A (97:3 H_2_O-methanol with 10 mM tributylamine adjusted to pH 8.2 by the addition of acetic acid to ∼10 mM final concentration). Solvent B was 100% methanol. Separation was achieved using the following gradient: 0 to 2.5 min, 5% B; 2.5 to 17 min, linear gradient from 5% B to 95% B; 17 to 19.5 min, 95% B; 19.5 to 20 min, linear gradient from 95% B to 5% B; 20 to 25 min, 5% B. Mass spectrometry parameters were full MS-SIM (single-ion monitoring) scanning between 70 and 1,000 m/z, automatic control gain (ACG) target of 1e6, maximum injection time (IT) of 40 ms, and resolution of 70,000 or 140,000 full width at half maximum (FWHM). Compounds were identified by retention time matching to pure standards and monoisotopic mass. Data were analyzed using the MAVEN software suite (81, 82). Metabolite mass isotopomer distributions (MIDs) from ^13^C labeling samples were corrected for ^13^C natural abundance using ElemCor (83), and the corrected MIDs were used throughout the text and graphs (see Table S4 in the supplemental material). ^2^H labeling data were not corrected for the natural abundance of ^2^H (∼0.02% abundance).

10.1128/mSystems.00736-19.8 Table S4 ^13^C and ^2^H labeling data in *C. thermocellum* and *T. saccharolyticum* corrected for ^13^C natural abundance (used in main figures and supplemental figures). Download Table S4, XLSX file, 0.1 MB. Copyright © 2020 Jacobson et al. 2020 Jacobson et al. This content is distributed under the terms of the Creative Commons Attribution 4.0 International license.

### Intracellular metabolite quantitation.

Intracellular metabolite concentrations were measured by growing E. coli RL3000 in medium containing solely [U-^13^C_6_]glucose and then extracting intracellular metabolites into solvent containing known concentrations of unlabeled standards for metabolites of interest. The solvent containing ^13^C-labeled intracellular metabolites and unlabeled standards was analyzed by LC-MS, and the ratio between the ^13^C and ^12^C peak intensities was used to determine intracellular metabolite concentrations (84). E. coli grown with [U-^13^C_6_]glucose was also extracted into solvent without standards, which was mixed in 1:5, 1:1, and 5:1 proportions with extracts from either *C. thermocellum* grown on cellobiose or *T. saccharolyticum* grown on glucose. The mixed samples were analyzed by LC-MS, and the ratio between labeled and unlabeled metabolite signals, combined with the calculated concentrations in E. coli, were used to measure metabolite concentrations in *C. thermocellum* and *T. saccharolyticum.* Intracellular metabolite quantitation was performed in biological triplicate.

### Aniline derivatization.

To enhance chromatographic separation and detection of some metabolites, we performed an aniline derivatization protocol for some samples used for metabolite quantitation (46). After the extraction of metabolites as described above, 100 μl extract was dried under N_2_ and then reconstituted in water. Ten microliters *N*-(3-dimethylaminopropyl)-*N*′-ethylcarbodiimide hydrochloride (EDC) solution and 10 μl 6 M aniline solution was added. Samples were vortexed for 2 h, and then 5 μl triethylamine was added to stop the reaction. Samples were centrifuged to remove debris and then subjected to LC-MS analysis as described above.

### Positional labeling in pyruvate and acetyl group labeling.

Positional labeling in pyruvate was determined by comparing the labeling patterns in ornithine (Orn) with those in acetylornithine (AcOrn). These compounds differ by a single acetyl group, received from acetyl-CoA, which is itself produced by decarboxylation of pyruvate. Thus, the acetyl group always contains only the 2nd and 3rd carbons of pyruvate, and the gain of labeled carbons by Orn during acetylation to AcOrn can be used as a readout of the position of labeled carbons in pyruvate. Acetyl group labeling was also measured by comparing the labeling patterns of glucosamine-6-phosphate to those in *N*-acetyl-glucosamine-6-phosphate.

### CO_2_ labeling.

During ^13^C labeling, unlabeled CO_2_ may come from two sources: (i) glucose dissimilation and (ii) carryover from inoculation. For glucose dissimulation, in cultures fed an equimolar mixture of [U^13^C_6_]glucose and unlabeled glucose, ∼50% of CO_2_ released during glucose fermentation to ethanol or acetate is expected to be unlabeled; the decarboxylation of pyruvate to form acetyl-CoA likely releases most of the cell-produced CO_2_. In cultures fed [1-^13^C_1_]- or [6-^13^C_1_]glucose, almost all CO_2_ released by the culture would be unlabeled. The ^13^C carbon under these conditions becomes the methyl carbon of pyruvate (Fig. 2), leaving the carboxyl carbon released by decarboxylation unlabeled. For carryover from inoculation, cultures were inoculated from a preculture containing unlabeled carbon sources, allowing unlabeled CO_2_ to be produced in the preculture and transferred to the working culture by syringe during inoculation.

To determine the labeling of intracellular CO_2_, we compared the labeling patterns of Orn with those of citrulline (Citr), which differs from Orn by incorporation of a single CO_2_ molecule. Thus, the difference between the labeling patterns of the two metabolites is due to the labeling of CO_2_. We used CO_2_ labeling derived this way solely to assist in our qualitative analysis of metabolism; CO_2_ was not assigned an MID during MFA.

### THF intermediate and mock purine labeling.

THF intermediate labeling was measured by comparing the labeling patterns of threonine (Thr) to the labeling pattern of methionine (Met). Met is formed from threonine by the addition of a single carbon from 5-methyltetrahydrofolate (MTHF), derived from the same pool as FTHF. Thus, the difference between Thr and Met labeling allowed us to determine FTHF labeling. Labeling of “mock purine” was produced by combining the labeling patterns of all precursors to purine formation, ribose-5-phosphate (R5P), glycine (gly), 2 THF intermediates, and CO_2_, into a single hypothetical MID for purines. This simulated MID showed agreement with measured labeling of purines ATP and GTP (Fig. S3C).

### Metabolic network model construction.

The *T. saccharolyticum* metabolic model was constructed from the KEGG genome-scale metabolic model, manually curated (10, 12, 22, 23) (Table S5A). The model contains the major central metabolic reactions from the EMP, nonoxidative pentose phosphate, and TCA pathways, along with fermentative, amino acid biosynthetic, and biomass-forming reactions. We constructed models containing alternative versions of pathways (i.e., an Entner-Doudoroff glycolytic pathway in addition to or instead of EMP glycolysis, a complete oxidative TCA cycle, or an oxidative PPP) to confirm that these pathways lacked activity in *T. saccharolyticum.* Our largest additions to the final genome-scale model were the inclusion of a complete set of reactions producing serine from 3-phosphoglycerate, gap filling of the citramalate pathway for isoleucine biosynthesis, and addition of an SBP bypass for the PPP, all of which were supported by qualitative analysis of steady-state labeling experiments and better model fits.

10.1128/mSystems.00736-19.9 Table S5 Reaction network and atom transitions used for MFA in *T. saccharolyticum* and *C. thermocellum*. Download Table S5, XLSX file, 0.02 MB. Copyright © 2020 Jacobson et al. 2020 Jacobson et al. This content is distributed under the terms of the Creative Commons Attribution 4.0 International license.

The *C. thermocellum* metabolic model was constructed using published metabolic network reconstructions, combined with KEGG annotations and manual curation (12, 18, 20–24, 26) (Table S5B). The model contains the major central metabolic reactions from the EMP, nonoxidative pentose phosphate, and TCA pathways, along with fermentative, amino acid biosynthetic, and biomass-forming reactions.

### Metabolic flux analysis.

MFA was performed using the INCA software suite (63). INCA is implemented in Matlab and simulates isotopic distributions according to the elementary metabolite unit (EMU) framework (64). We estimated intracellular fluxes by solving a nonlinear least-squares regression problem that minimizes the variance-weighted sum of squared residuals (SSR) between simulated and measured isotopic distributions of intracellular and extracellular metabolites. We combined all tracer data sets, together with uptake, excretion, and growth rates, for each organism into a single, statistically acceptable flux map using the COMPLETE-MFA technique (85). Labeling data from ^13^C and ^2^H tracer experiments were entered into INCA without correction for naturally abundant heavy isotopes (Table S6). We restarted flux estimation 25 times using random initial parameters to ensure a global SSR minimum had been reached. Reversible reactions were modeled as a forward and backward reaction. Net fluxes (*J*_net_) equals forward flux (*J^+^*) minus backward flux (*J^−^*), and exchange flux (*J*_exch_) equals min(*J^+^*, *J^−^*). Using the optimal solution, we calculated 95% confidence intervals for all estimated fluxes by performing a parameter continuation, which varies each flux to determine how sensitive the optimal SSR is to that flux. Upper and lower bounds are assigned to each flux by finding how far they can be varied before the SSR is perturbed past a critical point, corresponding to a chi-square distribution with a single degree of freedom.

10.1128/mSystems.00736-19.10 Table S6 ^13^C and ^2^H labeling data in *C. thermocellum* and *T. saccharolyticum* used for MFA analysis. Download Table S6, XLSX file, 0.05 MB. Copyright © 2020 Jacobson et al. 2020 Jacobson et al. This content is distributed under the terms of the Creative Commons Attribution 4.0 International license.

Some metabolites, such as pyruvate, malate, and fumarate, were present in both the cytosol and fermentation broth but did not appear to be taken up and used by the cell, just excreted over time. To account for the extracellular fractions of these metabolites, we modeled them in such a way as to allow dilution with their naturally labeled equivalents without allowing other reactions to access the naturally labeled fractions of these metabolites. Dilution is also present due to the incorporation of unlabeled atmospheric CO_2_, which was modeled by allowing intracellular CO_2_ to exchange with a pool of unlabeled CO_2._ We used a previously published biomass equation (86) and ethanol excretion rate (26), together with our measured uptake and excretion fluxes for lactate, acetate, glucose, and formate in *C. thermocellum.* The remaining glucose consumption was assigned to pyruvate, valine, asparagine, and alanine proportional to previous reported amounts (87).

Measured uptake and excretion fluxes for biomass, lactate, ethanol, acetate, and glucose in *T. saccharolyticum* were assigned based on previously reported end product titers (10, 39). Our analysis was unable to fully resolve the contributions of Ppdk versus the malate shunt regarding PEP-to-pyruvate flux. We therefore constructed two versions of the *C. thermocellum* model, whose only difference was the inclusion or omission of malic enzyme, and compared glycolytic flux ratios (*J^+^/J*^−^) and the derived free energies across them. We found a <1.0% cumulative difference in free energy of glycolytic reactions between the two models, with the single most affected reaction having only a 5.5% difference in free energy. Thus, the omission of malic enzyme had a negligible effect on *ΔG* measurements, and this model was used for all flux and free energy calculations presented in the text and figures.

### Goodness-of-fit analysis.

A χ^2^ test was used to determine whether the estimated fluxes adequately describe the measured data. The optimized SSR of a correct model and data set is a variable with a χ^2^ distribution with degrees of freedom equal to the number of fitted measurements (*n*) minus the number of estimated independent parameters (*p*). Fitted measurements are fitted external fluxes, namely, uptake, excretion, growth rates, and all non-zero MIDs. Estimated parameters are all free fluxes, including net, exchange, and dilution fluxes. We required that our models pass the χ^2^ test with a critical threshold of 0.05 (95% confidence), meaning the optimized SSR fell between $\chi_{\alpha /2}^{2}(n-p)$ and $\chi_{1-\alpha /2}^{2}(n-p)$. For *C. thermocellum*, the acceptable SSR range was 422.1 to 543.7, and the SSR after convergence was 499.2. For *T. saccharolyticum*, the acceptable SSR range was 434.3 to 557.5, and the SSR after convergence was 507.3.

### Estimation of Gibbs free energies.

Reaction free energies were calculated from MFA-derived flux measurements using the relation *ΔG = −RT* ln(*J^+^/J^−^*). For two reactions (Pfk and Pyk) in the glycolytic pathway of *T. saccharolyticum*, the 95% confidence interval lower bound for the reverse flux was 0, implying the reaction free energy is unbounded in the negative direction (i.e., the free energy is determined to be less than a discrete value with no lower bound). For these reactions, we supplemented our flux-based *ΔG* values with those calculated from metabolite concentrations using the relation *ΔG = ΔG°*′ *+ RT* ln*Q*, where *Q* is the ratio of the concentrations of products to reactants, *R* is the gas constant, and *T* is temperature in kelvin. Reaction standard free energies (*ΔG°*′) were retrieved from the online tool eQuilibrator, using a pH of 7 and ionic strength of 0.1 as settings (50, 88, 89). Metabolite concentrations provided a tighter bound on reaction free energies. The published reaction free energies for anaerobically grown E. coli (29) combine Pgi and Pfk as a single lumped reaction (Pgi-Pfk), so we supplemented these values with the free energy of Pgi and Pfk calculated from our measured intracellular metabolite concentrations for display (Fig. S4).
