# Supplementary figures and images for: In Vivo Thermodynamic Analysis of Glycolysis in Clostridium thermocellum and Thermoanaerobacterium saccharolyticum Using 13C and 2H Tracers
Source: mSystems. 2020 Mar 17;5(2):e00736-19. doi: 10.1128/mSystems.00736-19 (PMC7380578; doi:10.1128/mSystems.00736-19)

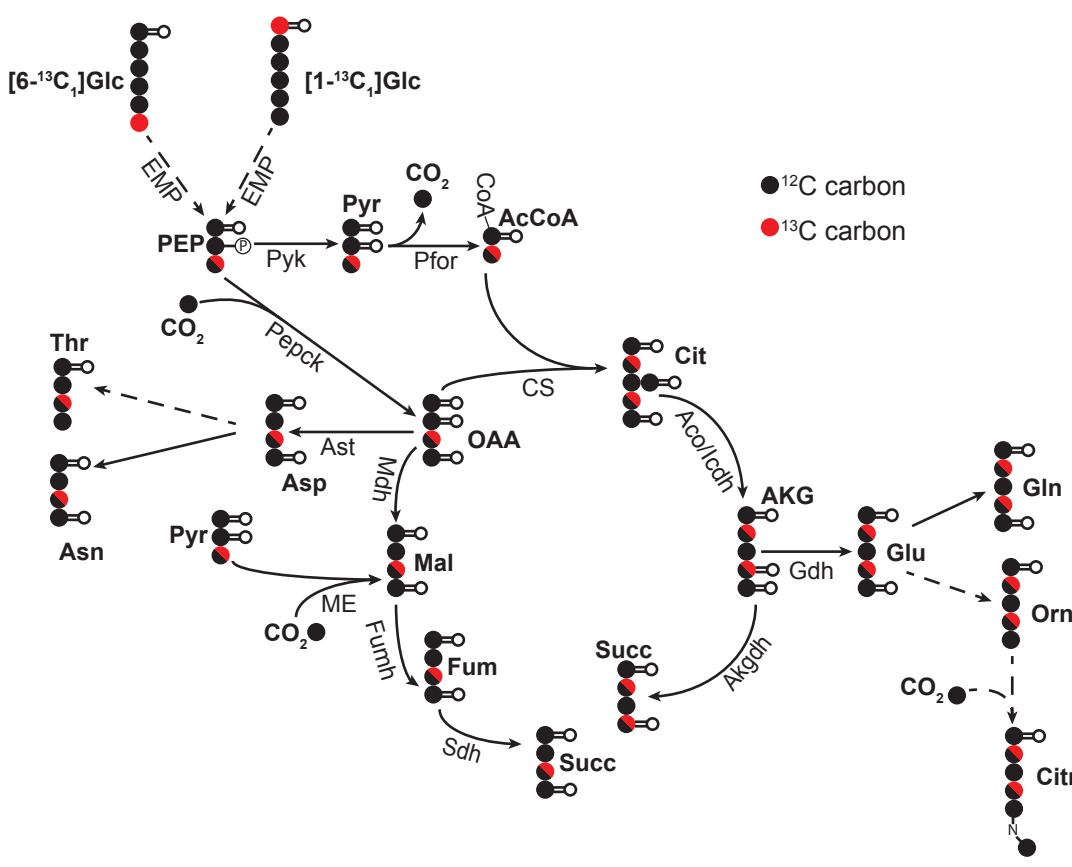

**A**

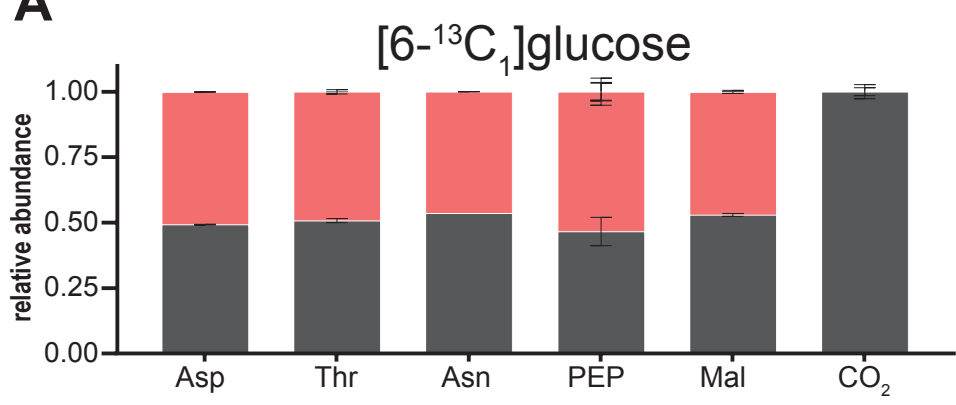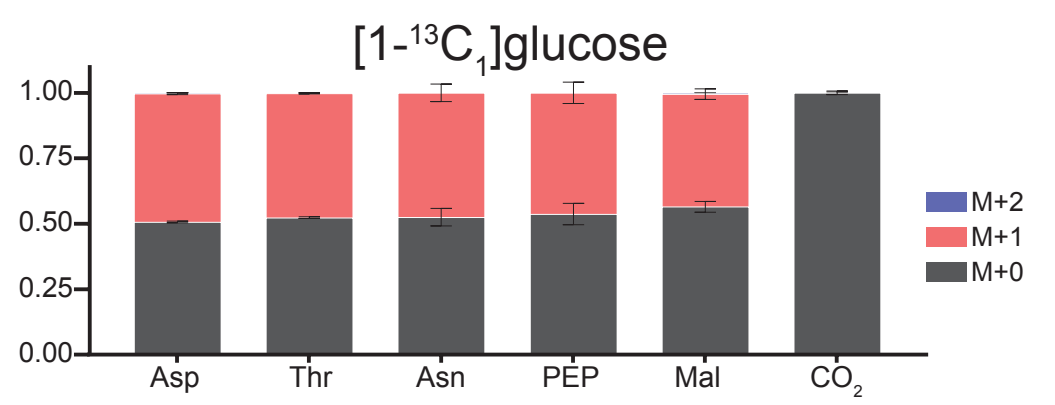

**B**

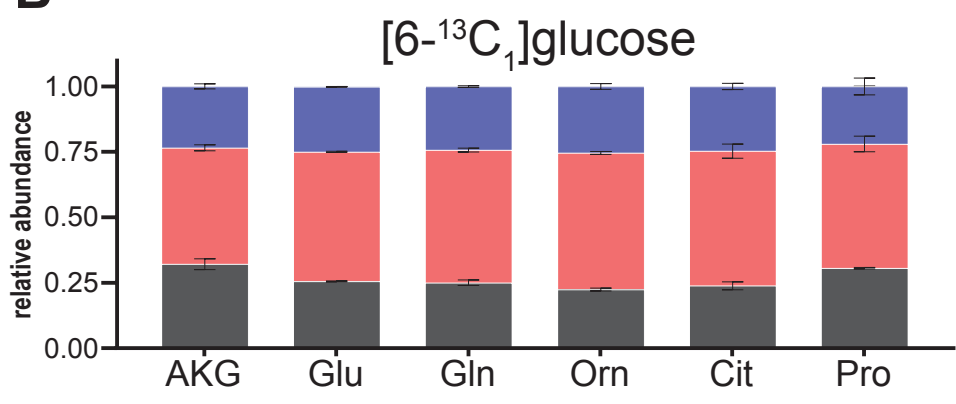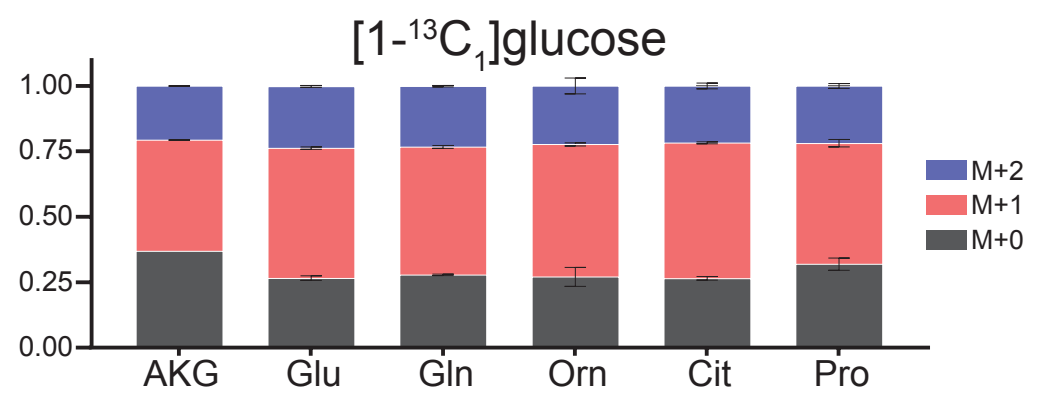

**C**

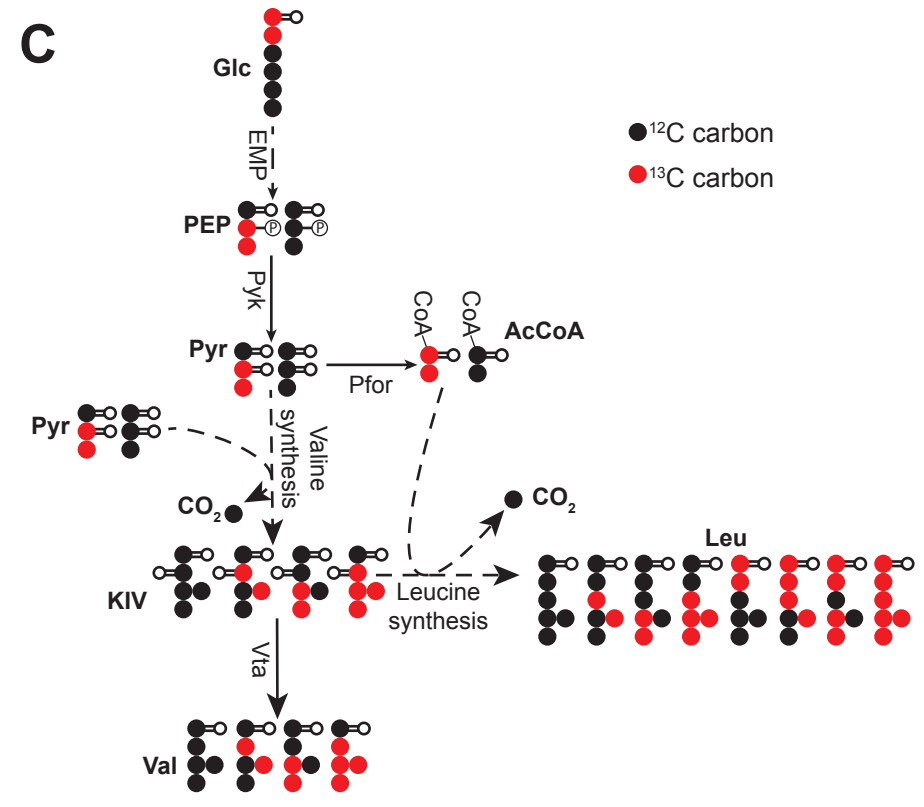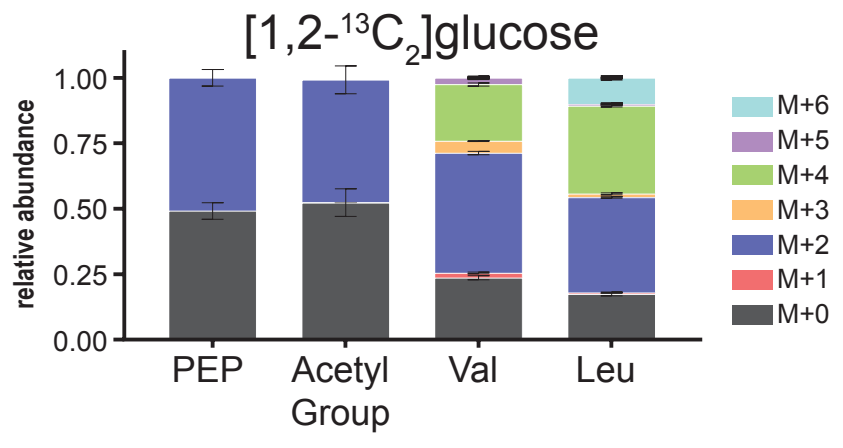

**D**

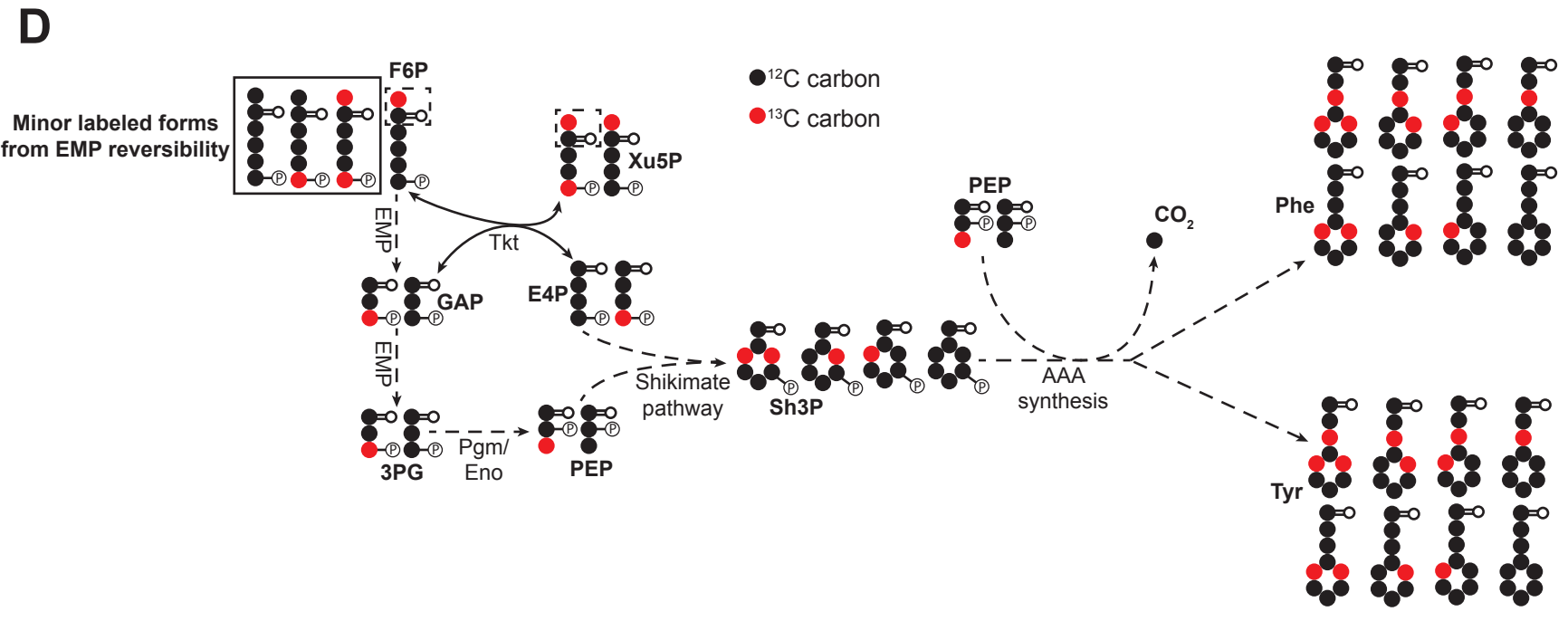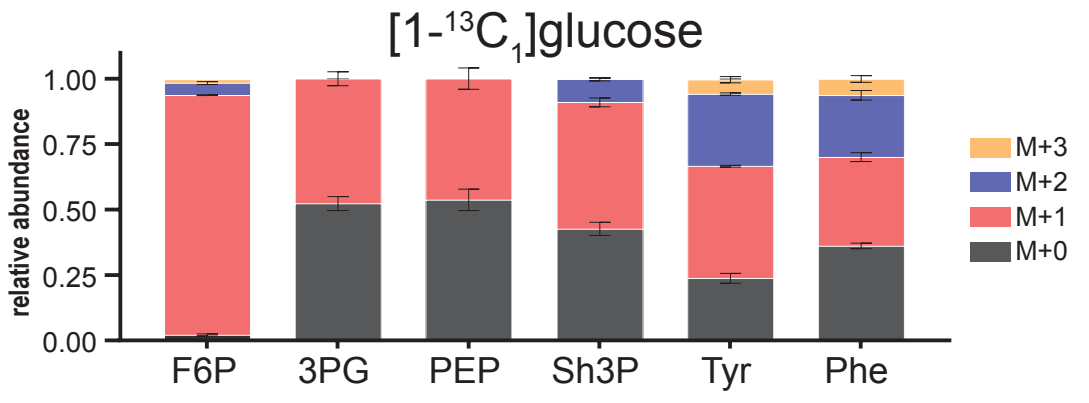

Supplement: FIG S2 [file mSystems.00736-19-sf002.pdf]

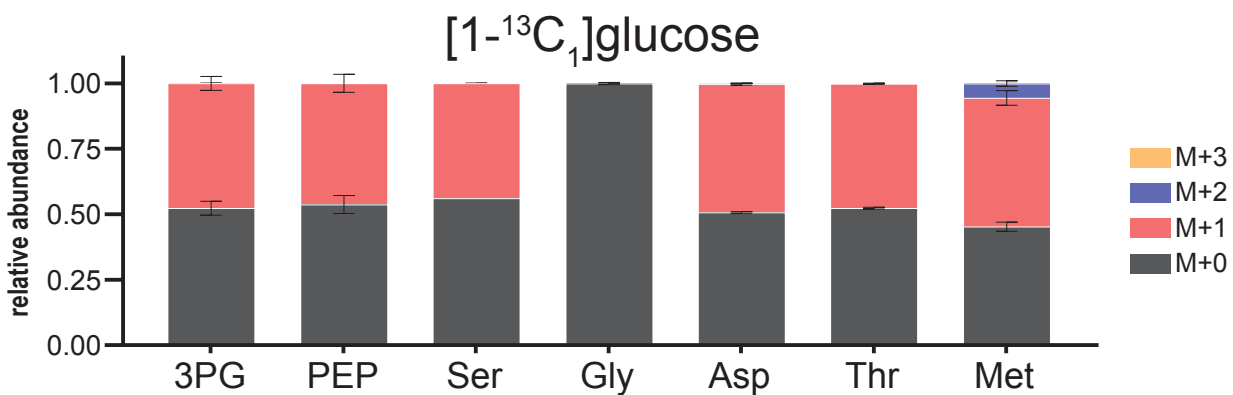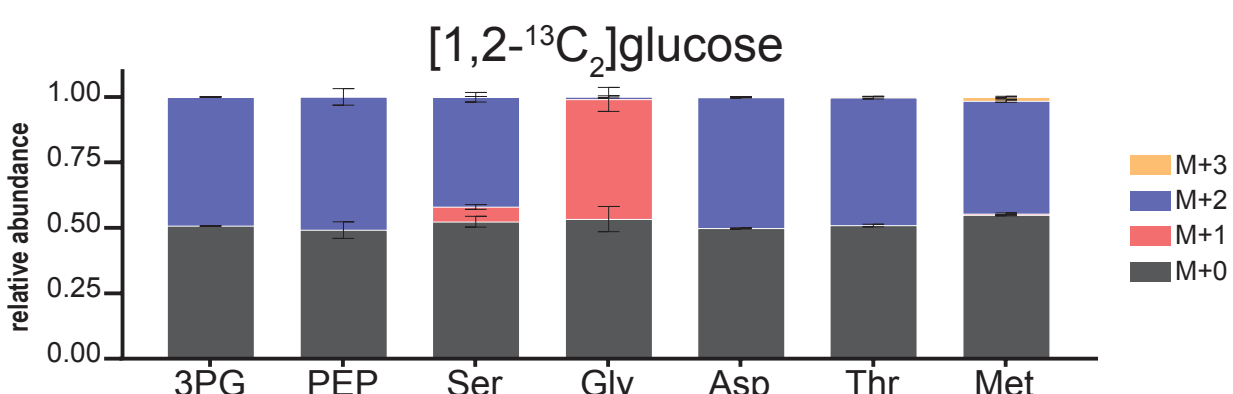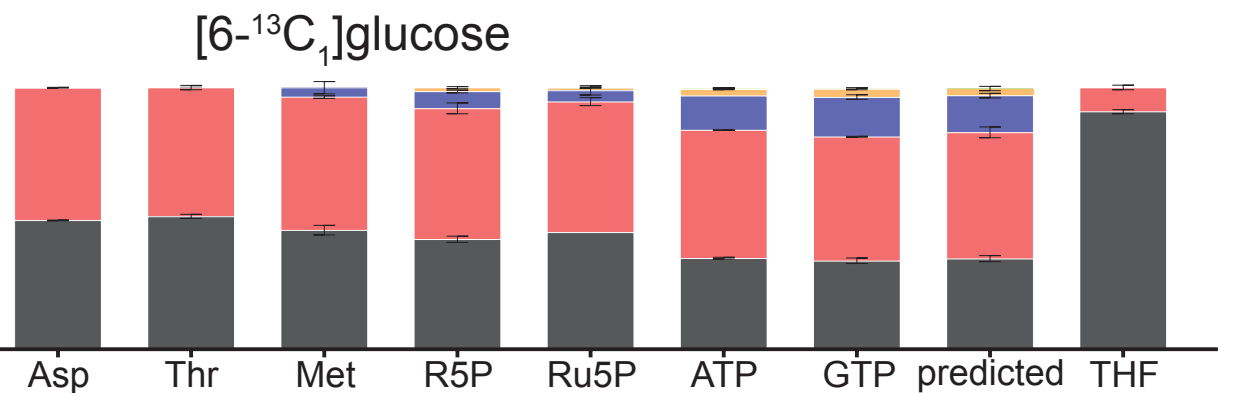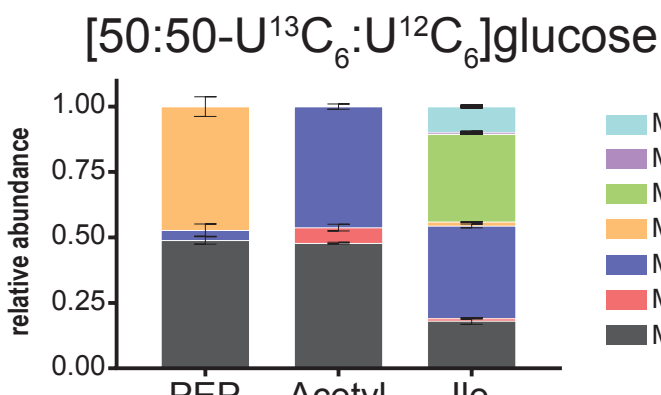

Supplement: FIG S3 [file mSystems.00736-19-sf003.pdf]
