# Supplementary material for: In Vivo Thermodynamic Analysis of Glycolysis in Clostridium thermocellum and Thermoanaerobacterium saccharolyticum Using 13C and 2H Tracers
Source: mSystems. 2020 Mar 17;5(2):e00736-19. doi: 10.1128/mSystems.00736-19 (PMC7380578; doi:10.1128/mSystems.00736-19)
Supplement: FIG S4 [file mSystems.00736-19-sf004.pdf]

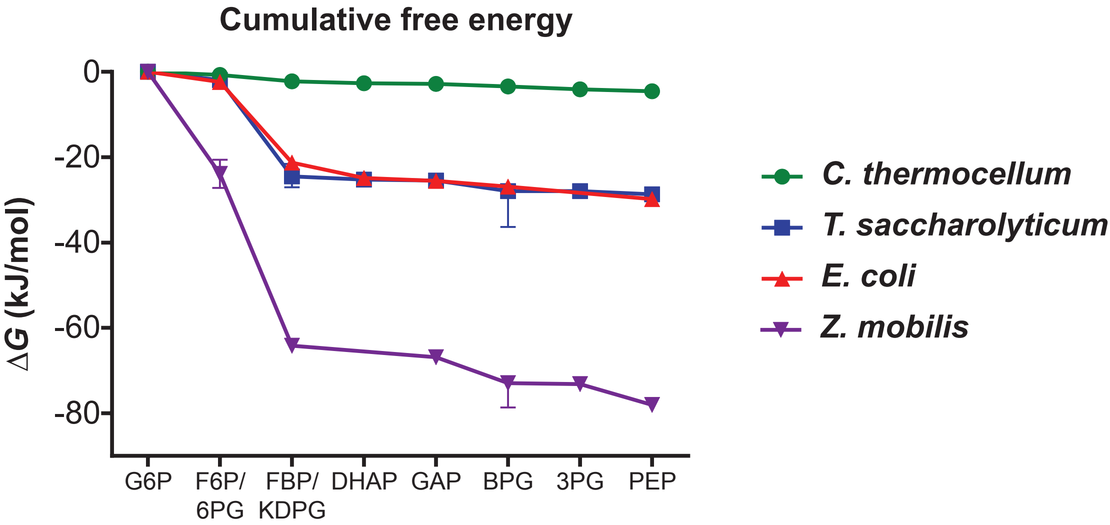

E. coli free energy values are adapted from Park *et. al.*, 2019
Z. mobilis free energy values are adapted from Jacobson *et. al.*, 2019
