# Supplementary material for: In Vivo Thermodynamic Analysis of Glycolysis in Clostridium thermocellum and Thermoanaerobacterium saccharolyticum Using 13C and 2H Tracers
Source: mSystems. 2020 Mar 17;5(2):e00736-19. doi: 10.1128/mSystems.00736-19 (PMC7380578; doi:10.1128/mSystems.00736-19)
Supplement: FIG S1 [file mSystems.00736-19-sf001.pdf]

A

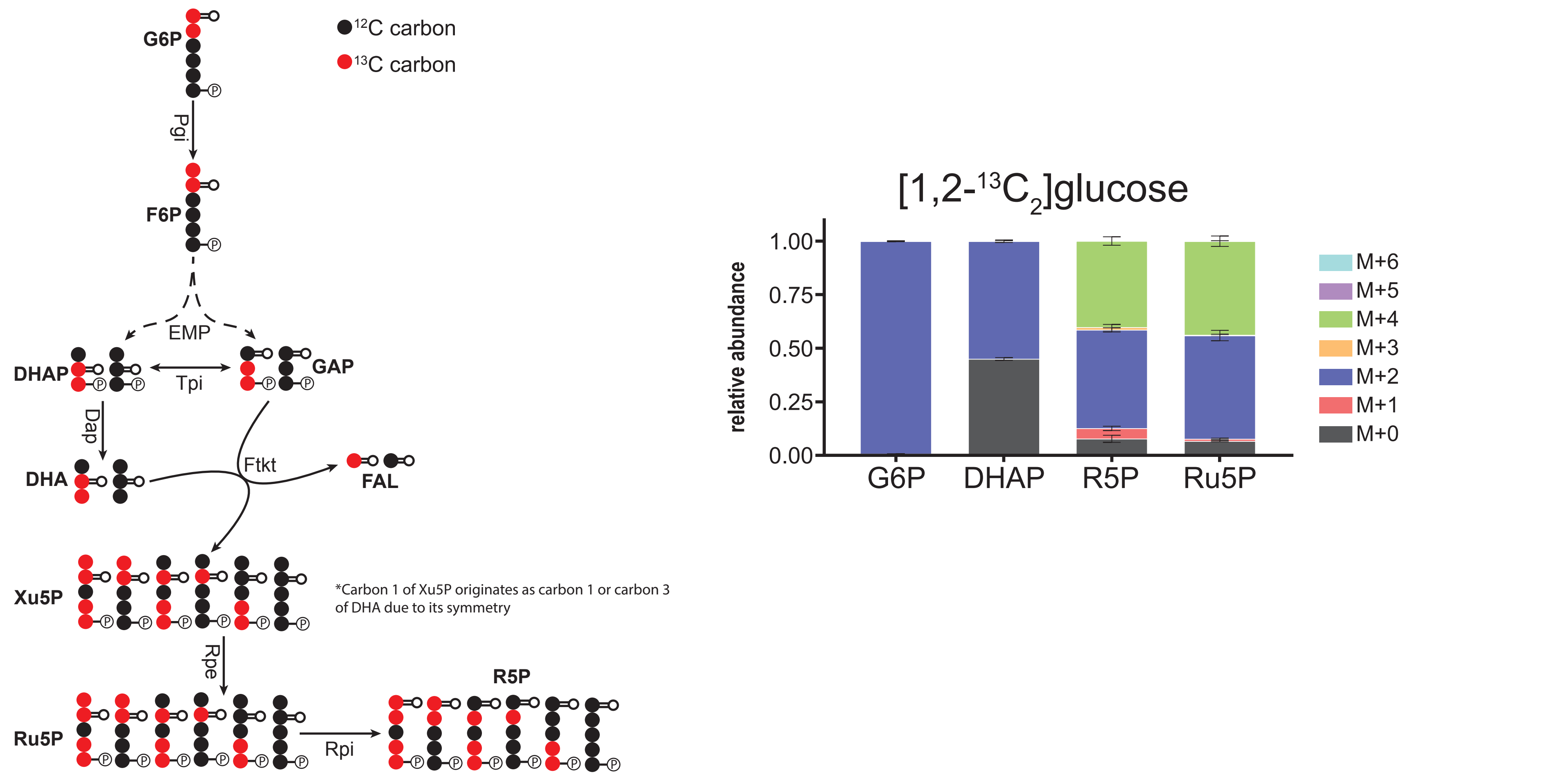

G6P
Pgi
F6P
EMP
DHAP
Tpi
GAP
Dap
DHA
Ftkt
FAL
Xu5P
*Carbon 1 of Xu5P originates as carbon 1 or carbon 3 of DHA due to its symmetry
Rpe
Ru5P
Rpi
R5P
12C carbon
13C carbon
[1,2-13C2]glucose
relative abundance
1.00
0.75
0.50
0.25
0.00
G6P
DHAP
R5P
Ru5P
M+6
M+5
M+4
M+3
M+2
M+1
M+0

B

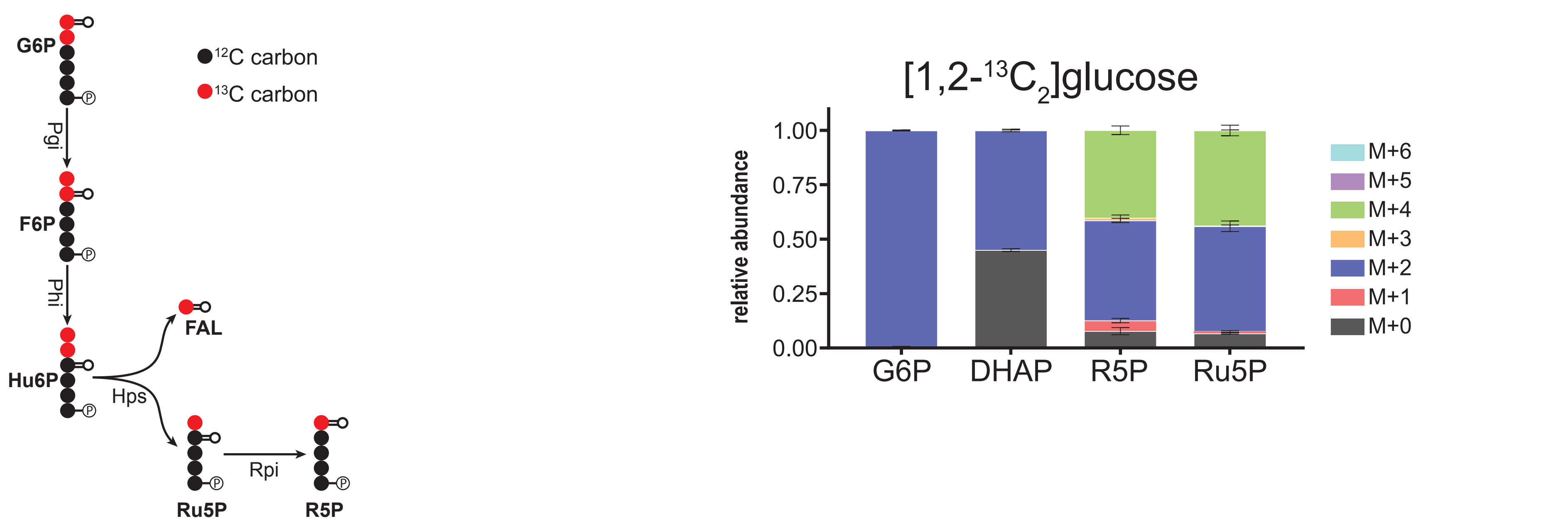

G6P
Pgi
F6P
Phi
Hu6P
Hps
FAL
Ru5P
Rpi
R5P
12C carbon
13C carbon
[1,2-13C2]glucose
relative abundance
1.00
0.75
0.50
0.25
0.00
G6P
DHAP
R5P
Ru5P
M+6
M+5
M+4
M+3
M+2
M+1
M+0

C

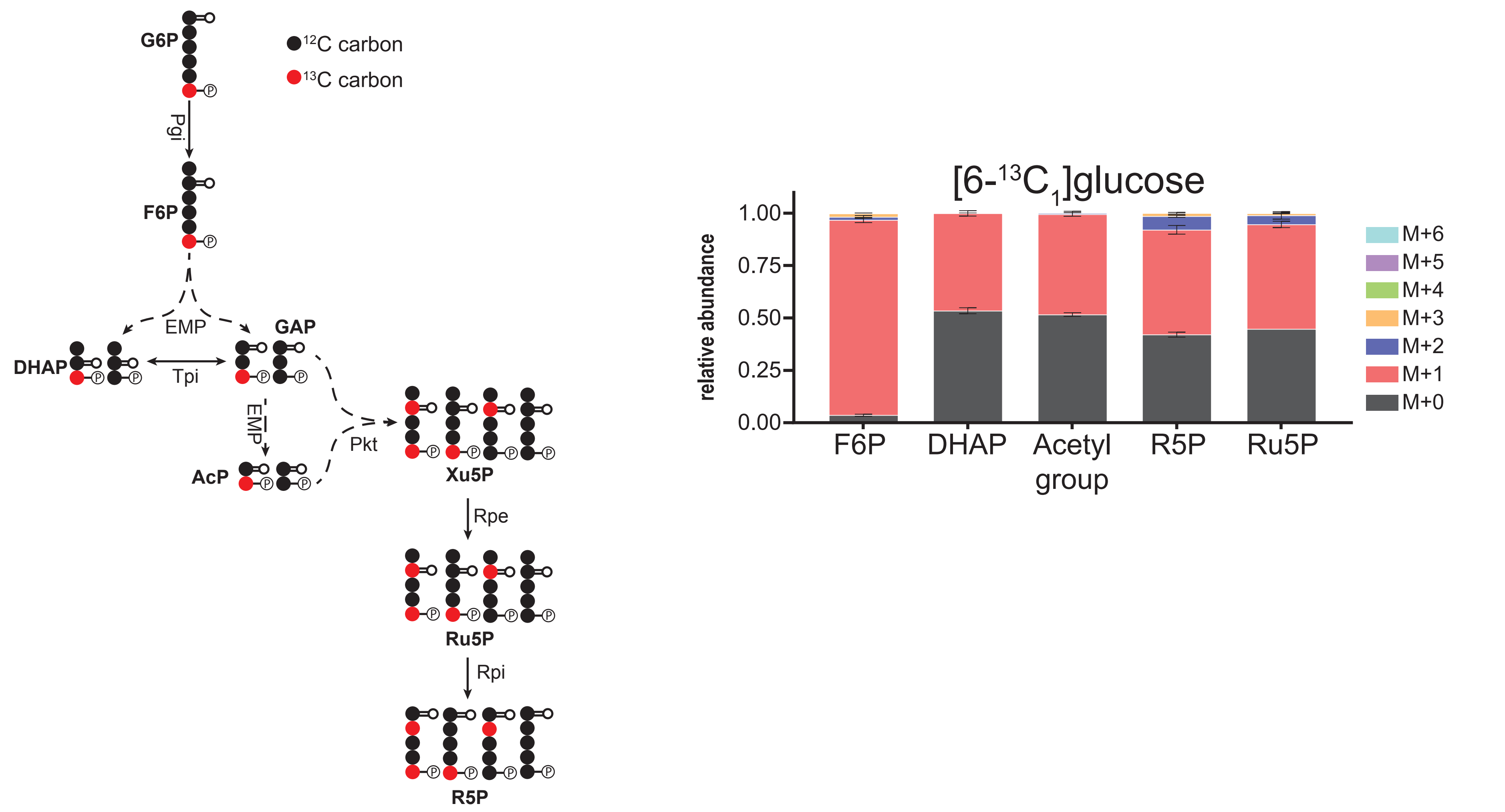

G6P
Pgi
F6P
EMP
DHAP
Tpi
GAP
EMP
AcP
Pkt
Xu5P
Rpe
Ru5P
Rpi
R5P
12C carbon
13C carbon
[6-13C1]glucose
relative abundance
1.00
0.75
0.50
0.25
0.00
F6P
DHAP
Acetyl group
R5P
Ru5P
M+6
M+5
M+4
M+3
M+2
M+1
M+0

D

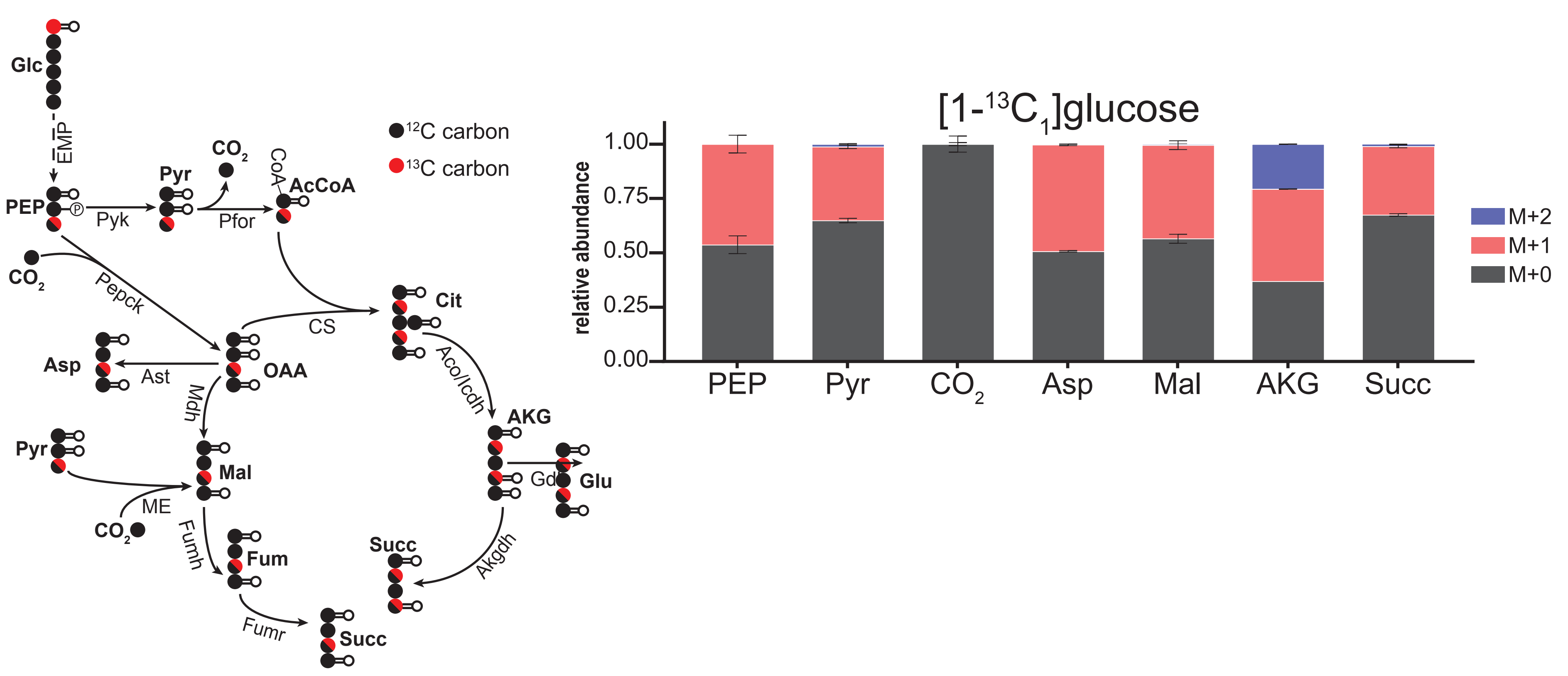

Glc
EMP
PEP
Pyk
Pyr
CO2
Pfor
CoA
AcCoA
CO2
Pepck
CS
Cit
Asp
Ast
OAA
Mdh
Aco/Icdh
AKG
Pyr
Mal
Gdh
Glu
ME
CO2
Fumh
Fum
Succ
Akgdh
Fumr
Succ
12C carbon
13C carbon
[1-13C1]glucose
relative abundance
1.00
0.75
0.50
0.25
0.00
PEP
Pyr
CO2
Asp
Mal
AKG
Succ
M+2
M+1
M+0

E

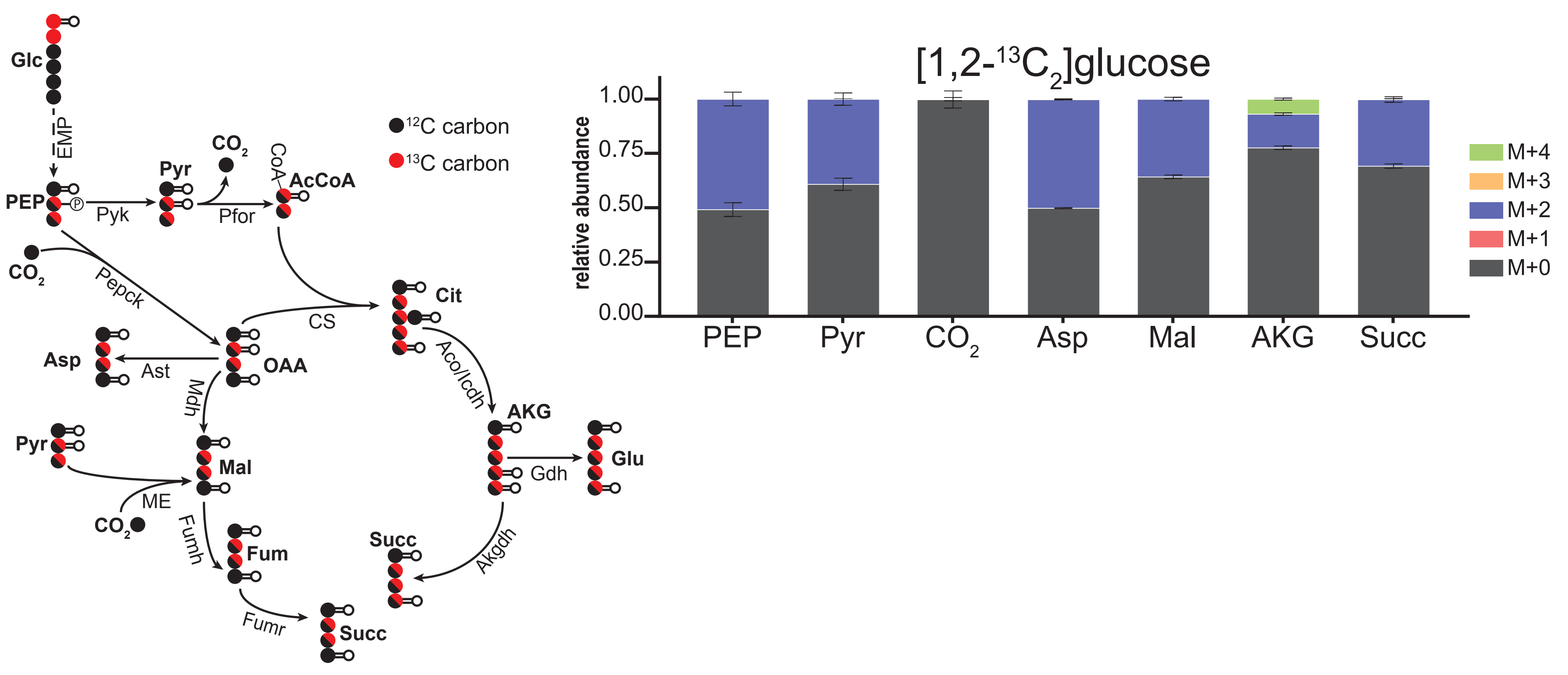

Glc
EMP
PEP
Pyk
Pyr
CO2
Pfor
CoA
AcCoA
CO2
Pepck
CS
Cit
Asp
Ast
OAA
Mdh
Aco/Icdh
AKG
Pyr
Mal
Gdh
Glu
ME
CO2
Fumh
Fum
Succ
Akgdh
Fumr
Succ
12C carbon
13C carbon
[1,2-13C2]glucose
relative abundance
1.00
0.75
0.50
0.25
0.00
PEP
Pyr
CO2
Asp
Mal
AKG
Succ
M+4
M+3
M+2
M+1
M+0
